# Supplementary material for: Fluorinated polyamidoamine dendrimer-mediated miR-23b delivery for the treatment of experimental rheumatoid arthritis in rats
Source: Nat Commun. 2023 Feb 20;14:944. doi: 10.1038/s41467-023-36625-7 (PMC9941585; doi:10.1038/s41467-023-36625-7)
Supplement: Supplementary file 1 — Supplementary Information [file 41467_2023_36625_MOESM1_ESM.pdf]

Supplementary Information

Fluorinated polyamidoamine dendrimer-mediated miR-23b delivery for the treatment of experimental rheumatoid arthritis

Haobo Han[1], Jiakai Xing[1], Wenqi Chen[1], Jiaxin Jia[1], Quanshun Li[1,*]

[1]*Key Laboratory for Molecular Enzymology and Engineering of Ministry of Education, School of Life Sciences, Jilin University, Changchun 130012, China*

*Corresponding author.

E-mail: quanshun@jlu.edu.cn.

Tel. and Fax: +86-431-85155200.

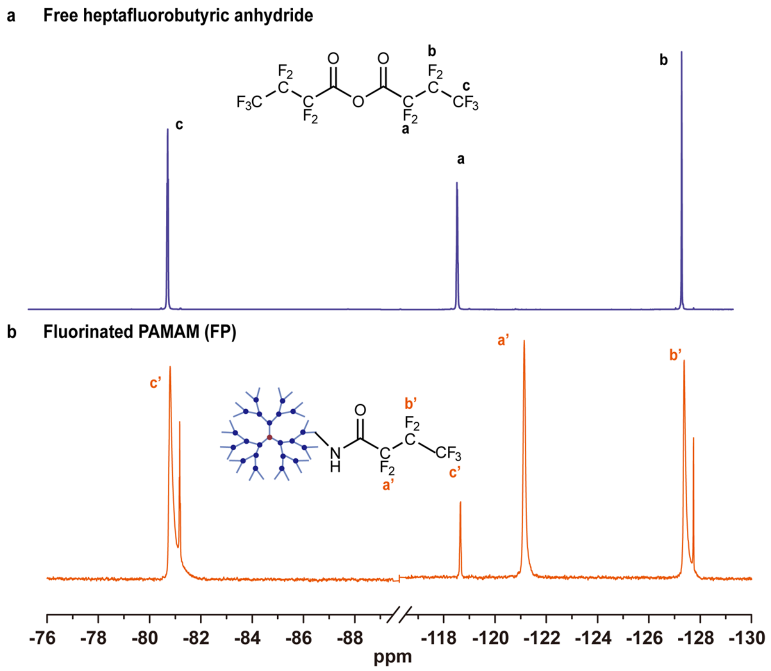

Supplementary Fig. 1. The structural characterization of fluorinated PAMAM (FP) using ^{19}F NMR. **(a)** Free heptafluorobutyric anhydride. **(b)** FP. The peak (a) in heptafluorobutyric anhydride significantly shifted to the peak (a’) in FP.

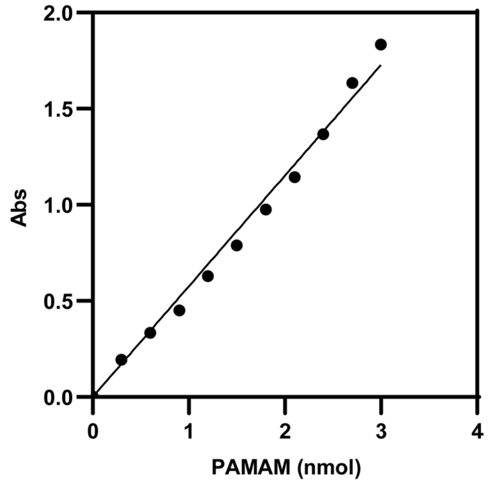

Supplementary Fig. 2. The number of fluorinated groups on the surface of FP was determined by the ninhydrin assay. The number of amines groups was calculated by the calibration curve (Abs = 0.5986x - 0.0475, R^2 = 0.9907, Abs represents the absorbance of sample at 570 nm, x was the concentration of primary amine groups, nmol). Three repeats were conducted for each sample.

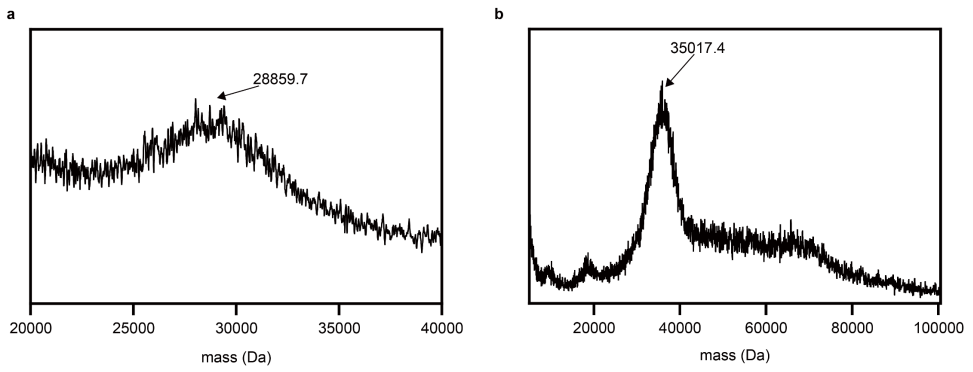

Supplementary Fig. 3. Molecular weight of PAMAM **(a)** and FP **(b)** measured using MALDI-TOF MS analysis.

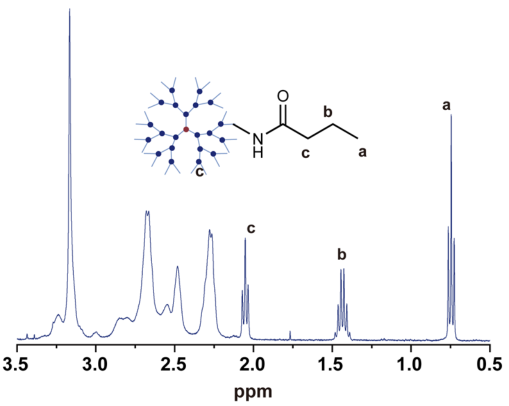

Supplementary Fig. 4. The characterization of butyric acid-modified PAMAM (termed as HP) using ^{1}H NMR. An average number of 28 butyric acids were calculated to be conjugated to each PAMAM molecule.

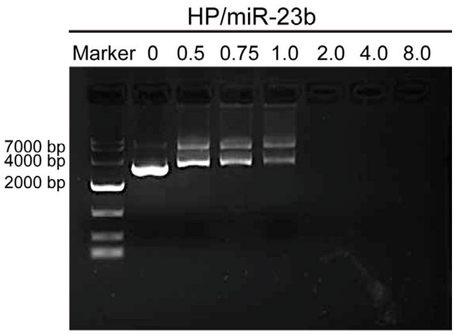

Supplementary Fig. 5. The binding and condensation ability of HP with miR-23b at different N/P ratios. Lane 1: Marker, Lane 2-8: HP/miR-23b nanoparticles at N/P ratios of 0, 0.5, 0.75, 1.0, 2.0, 4.0, and 8.0, respectively. A representative image of three biologically independent experiments from each group is shown.

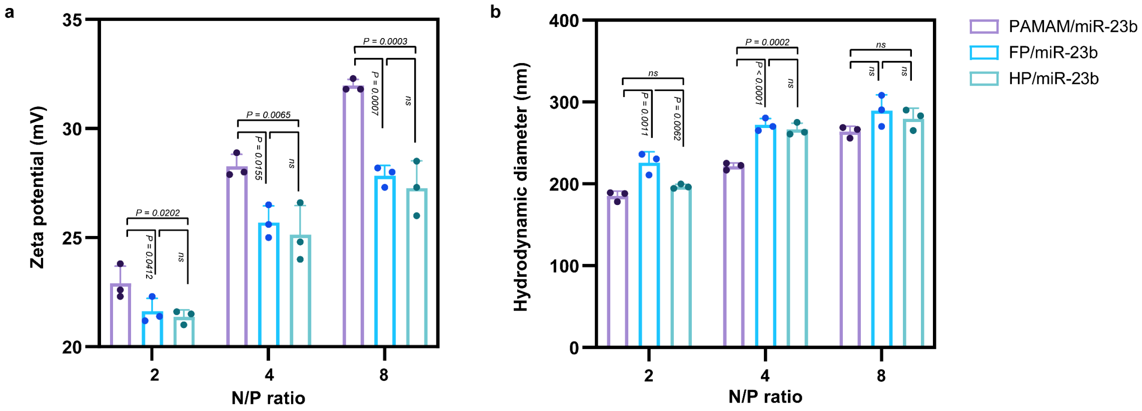

Supplementary Fig. 6. (a) Zeta potential and **(b)** hydrodynamic diameter of carrier/miR-23b nanoparticles. Data are presented as mean value ± SD (n= 3 independent experiments). Two-sided statistical analysis is measured by one-way ANOVA with LSD test.

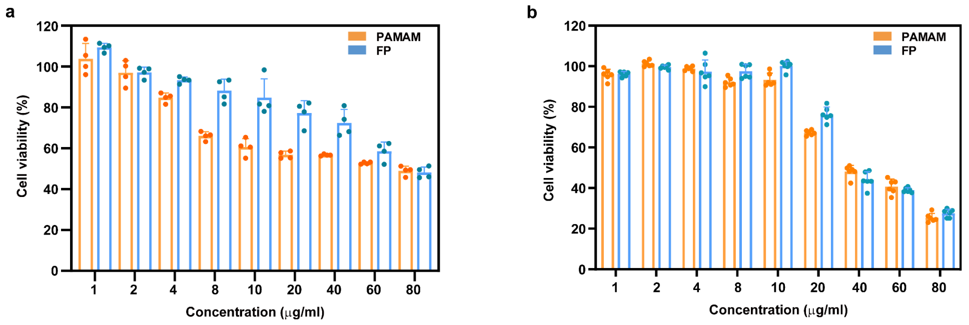

Supplementary Fig. 7. The cytotoxicity analysis of carriers in BMDMs **(a)** and RAW264.7 cells **(b)** for 48 h. Data are presented as mean value ± SD (n= 4 independent experiments for **a** and n =6 independent experiments for **b**).

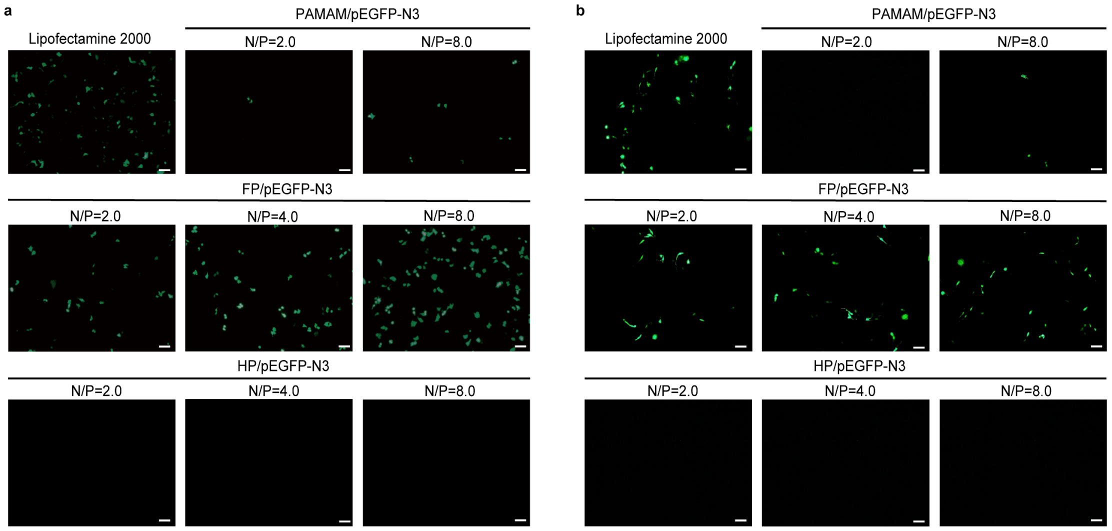

Supplementary Fig. 8. Fluorescence images of the carriers-mediated pEGFP-N3 transfection in HeLa cells **(a)** and NIH3T3 cells **(b)** for 48 h. Lipofectamine 2000 was served as the control. Scale bar: 100 µm. A representative image of three biologically independent experiments from each group is shown in **a** and **b**.

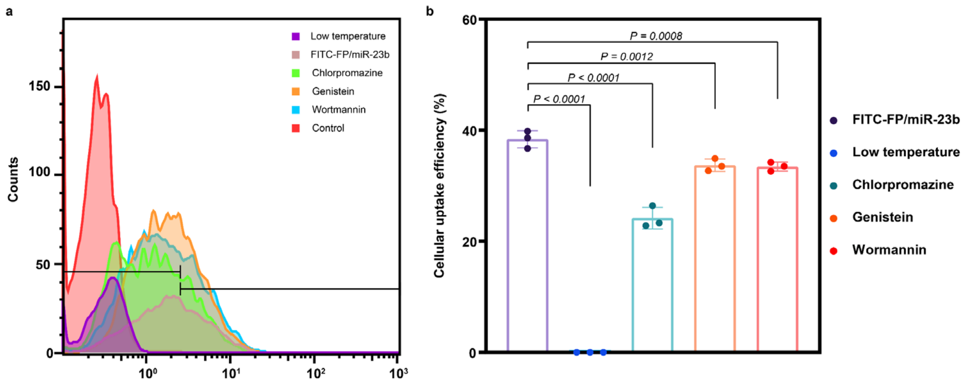

Supplementary Fig. 9. The analysis of endocytosis pathway using flow cytometry. The cells were transfected with FITC-FP/miR-23b nanoparticles (N/P ratio of 2.0) following the pre-treatment with different endocytic inhibitors for 30 min. **(a)** The flow cytometry analysis for the endocytosis of FITC-FP/miR-23b nanoparticles following the pre-treatment with endocytic inhibitors. **(b)** Cellular uptake efficiency of FITC-FP/miR-23b nanoparticles following the pre-treatment with endocytic inhibitors. Data are presented as mean value ± SD (n= 3 independent experiments). One-sided statistical analysis is measured by one-way ANOVA with LSD test.

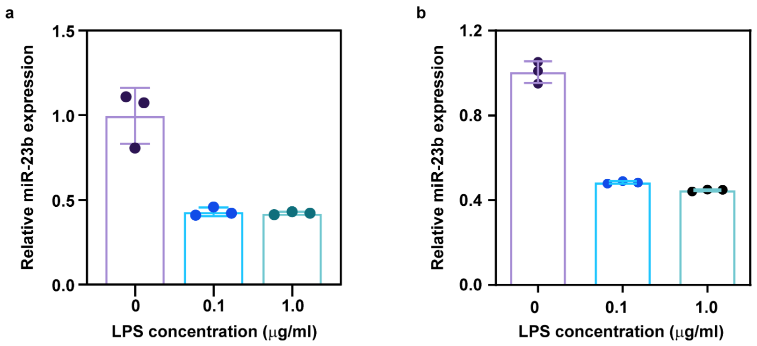

Supplementary Fig. 10. Relative miR-23b expression level in **(a)** BMDMs and **(b)** RAW264.7 cells after the treatment with LPS for 24 h measured by qPCR. Data are presented as mean value ± SD (n= 3 independent experiments). One-sided statistical analysis is measured by one-way ANOVA with LSD test.

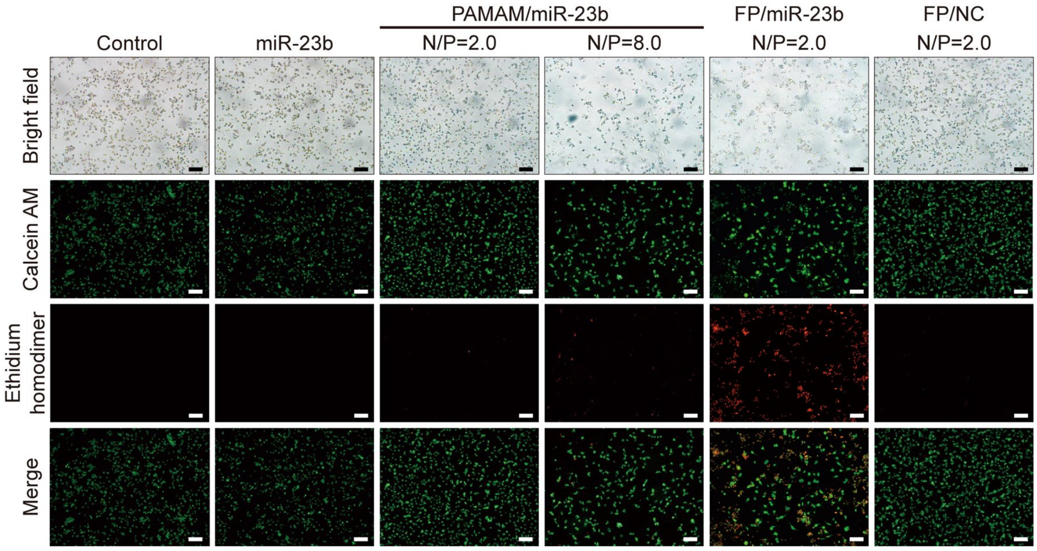

Supplementary Fig. 11. Live/dead cell staining of BMDMs cells after the miR-23b transfection. The live cells were labeled with green fluorescence owing to the enzymatic hydrolysis of calcein AM whereas the dead cells were stained with red fluorescence generated from the interaction of ethidium homodimer to DNA. Scale bar: 100 μm. A representative image of three biologically independent experiments from each group is shown.

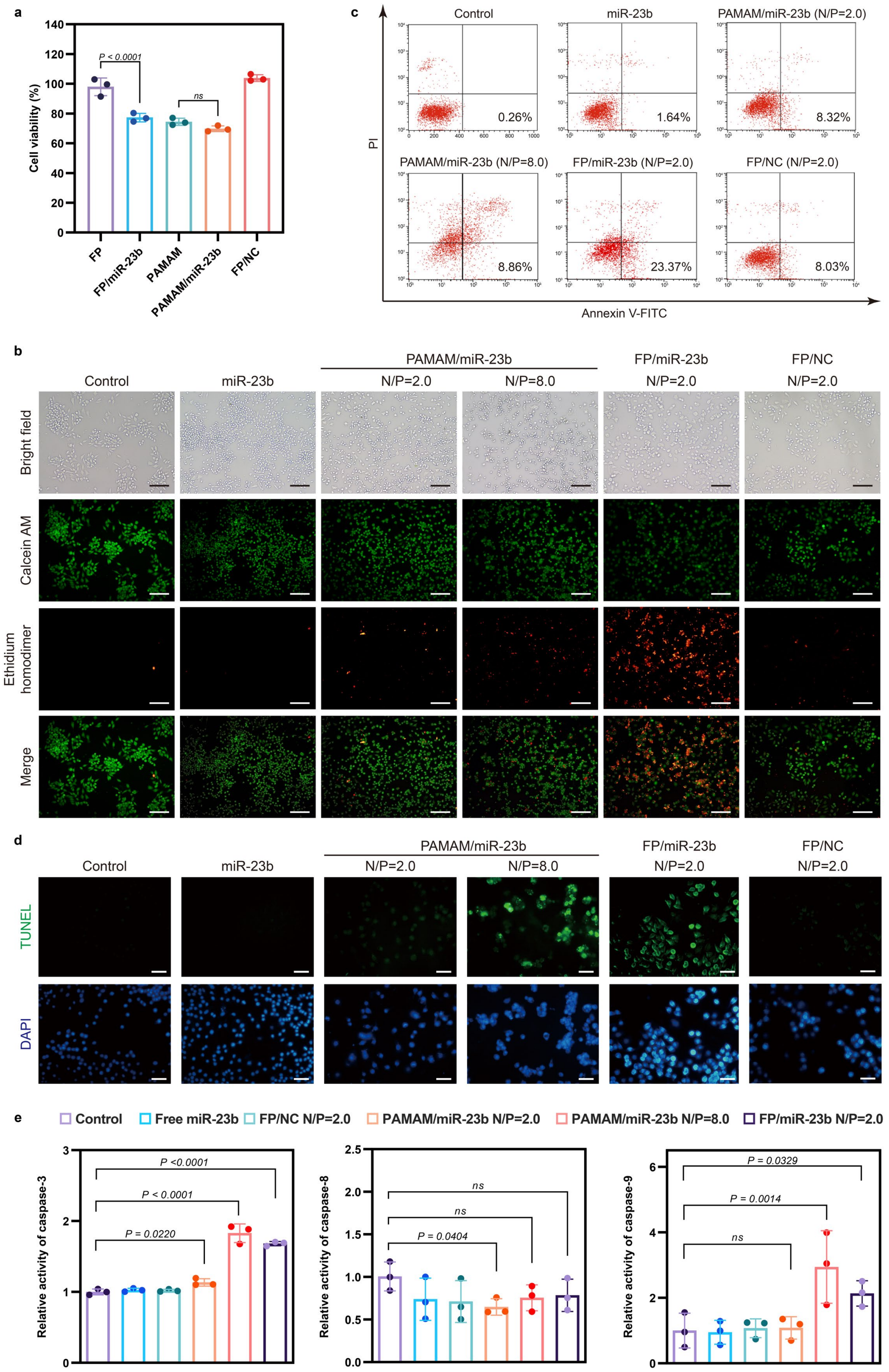

Supplementary Fig. 12. Inhibition of cell proliferation after miR-23b transfection in LPS-stimulated RAW264.7 cells. **(a)** The proliferative inhibition induced by the miR-23b transfection using MTT method. Data are presented as mean value ± SD of triplicate

experiments. One-sided statistical analysis is measured by one-way ANOVA with LSD test. **(b)** Live/dead cell staining of RAW264.7 cells after the miR-23b transfection. The live cells were labeled with green fluorescence owing to the enzymatic hydrolysis of calcein AM whereas the dead cells were stained with red fluorescence generated from the interaction of ethidium homodimer to DNA. Scale bar: 100 μm. A representative image of three biologically independent experiments from each group is shown. **(c)** The cell apoptosis analysis using the flow cytometry based on the Annexin V-FITC/PI staining, with apoptotic ratios of 0.26% in control group, 1.64% in free miR-23b group, 8.32% in PAMAM/miR-23b (N/P=2.0) group, 8.86% in PAMAM/miR-23b (N/P=8.0) group, 23.37% in FP/miR-23b (N/P=2.0) group and 8.03% in FP/NC (N/P=2.0) group, respectively (n= 3 independent experiments). **(d)** The apoptosis effect induced by miR-23b delivery visualized by TUNEL staining. Nuclei, blue (DAPI); TUNEL, green. Scale bar: 50 μm. A representative image of three biologically independent experiments from each group is shown. **(e)** Relative activity of caspase-3 (left), caspase-8 (middle) and caspase-9 (right) following the miR-23b transfection. Data are presented as mean value ± SD (n= 3 independent experiments). One-sided statistical analysis is measured by one-way ANOVA with LSD test.

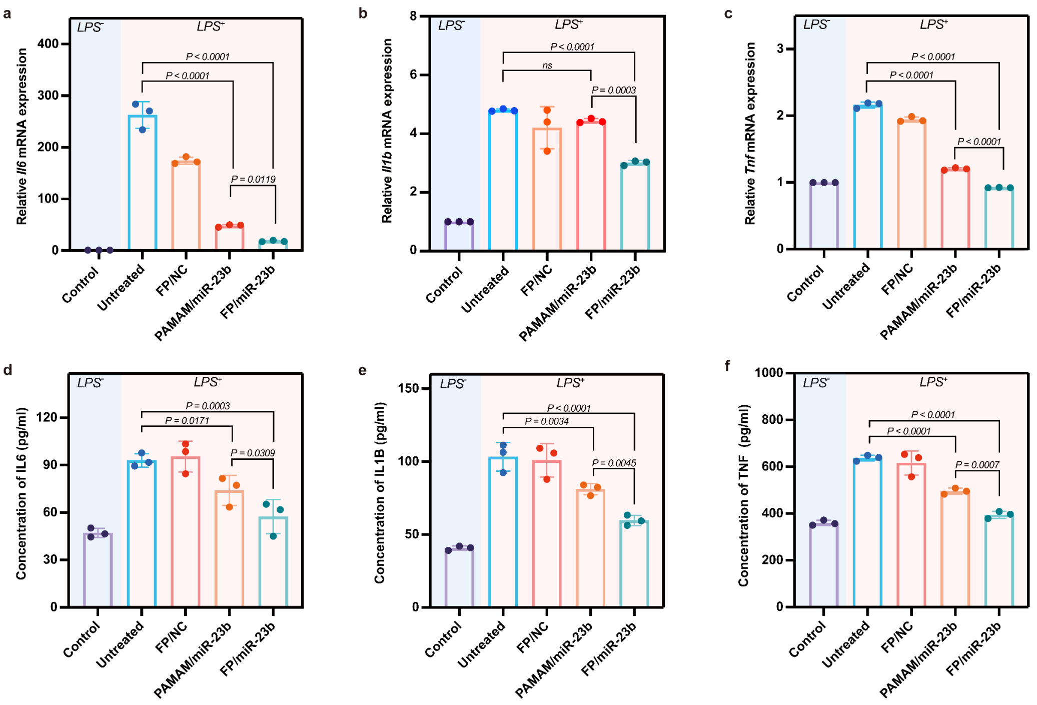

Supplementary Fig. 13. Inhibition of inflammation by miR-23b delivery in RAW264.7 cells. **(a-c)** Relative mRNA expression of pro-inflammatory cytokines *Il6* **(a)**, *Il1b* **(b)** and *Tnf* **(c)** in LPS-stimulated RAW264.7 cells after the miR-23b delivery. **(d-f)** Levels of IL6 **(d)**, IL1B **(e)** and TNF **(f)** in cell culture supernatants were determined by ELISA methods. In a-f, LPS^- represents the RAW264.7 cells without lipopolysaccharide stimulation, and LPS^+ groups represent the RAW264.7 cells treated with lipopolysaccharide (0.1 μg/ml) for 24 h. Data are presented as mean value ± SD (n= 3 independent experiments). One-sided statistical analysis is measured by one-way ANOVA with LSD test.

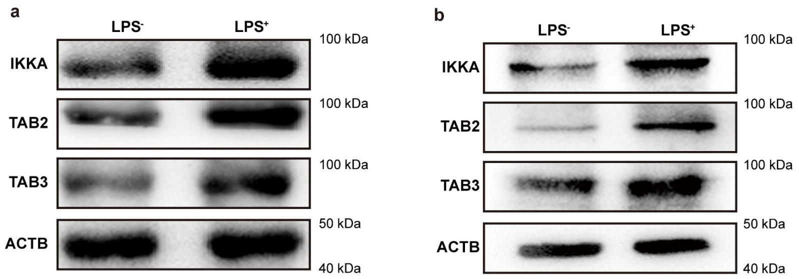

Supplementary Fig. 14. The expression level analysis of TAB2, TAB3 and IKKA in **(a)** BMDMs and **(b)** RAW264.7 cells after the LPS stimulation (0.1 μg/ml) for 24 h. LPS^- represents the untreated macrophages while LPS^+ represents the cells treated with lipopolysaccharide (0.1 μg/ml) for 24 h. A representative blot of three independent experiments is shown in **a** and **b**.

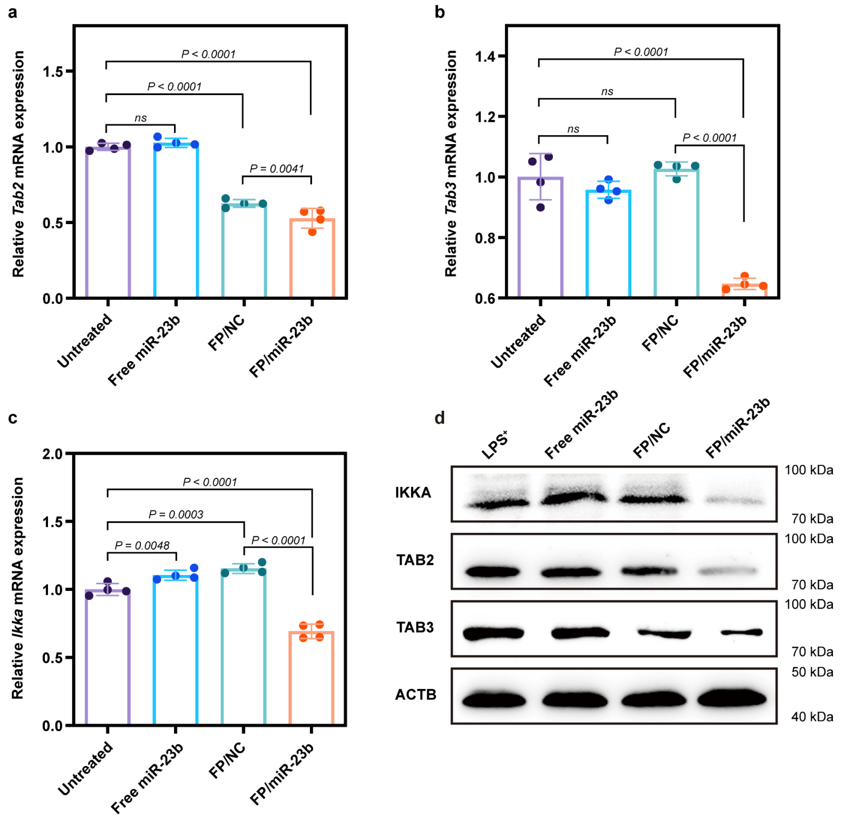

Supplementary Fig. 15. FP/miR-23b nanoparticles downregulated the miR-23b targets in LPS-stimulated RAW264.7 cells. **(a-c)** Relative mRNA expression of *Tab2* **(a)**, *Tab3* **(b)** and *Ikka* **(c)** in LPS-stimulated BMDMs cells after the miR-23b transfection. FP/miR-23b and FP/NC nanoparticles were prepared at N/P ratio of 2.0. PAMAM/miR-23b and FP/miR-23b (or FP/NC) nanoparticles were prepared at N/P ratios of 8.0 and 2.0, respectively. Data are presented as mean value ± SD (n= 3 independent experiments). One-sided statistical analysis is measured by one-way ANOVA with LSD test. (**d**) The protein expression level of TAB2, TAB3 and IKKA in LPS-stimulated RAW264.7 cells after the miR-23b transfection. LPS^+ group represents the RAW264.7 cells treated with lipopolysaccharide (0.1 μg/ml) for 24 h. A representative blot of three independent experiments is shown.

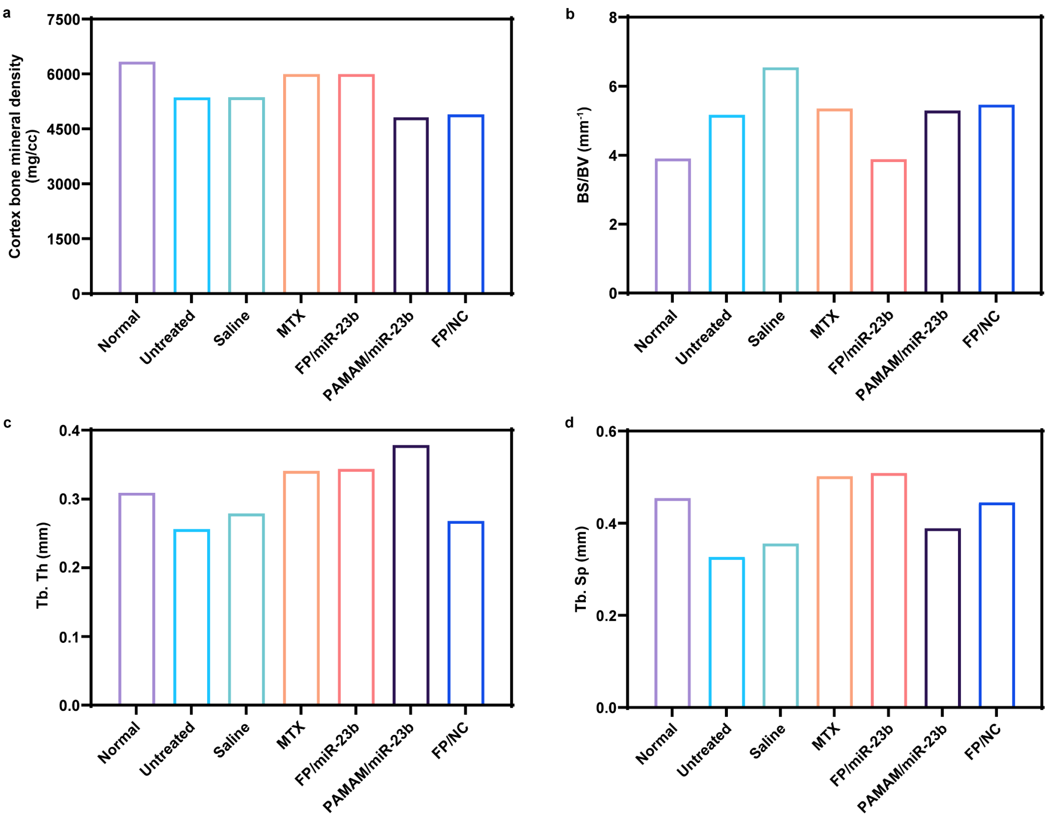

Supplementary Fig. 16. Quantitative bone parameters obtained from micro-CT in AIA rats. **(a)** Cortex bone mineral density (BMD), **(b)** bone surface/bone volume (BS/BV), **(c)** trabecular thickness (Tb. Th) and **(d)** trabecular spacing (Tb. Sp). Data are presented as single values from each group.

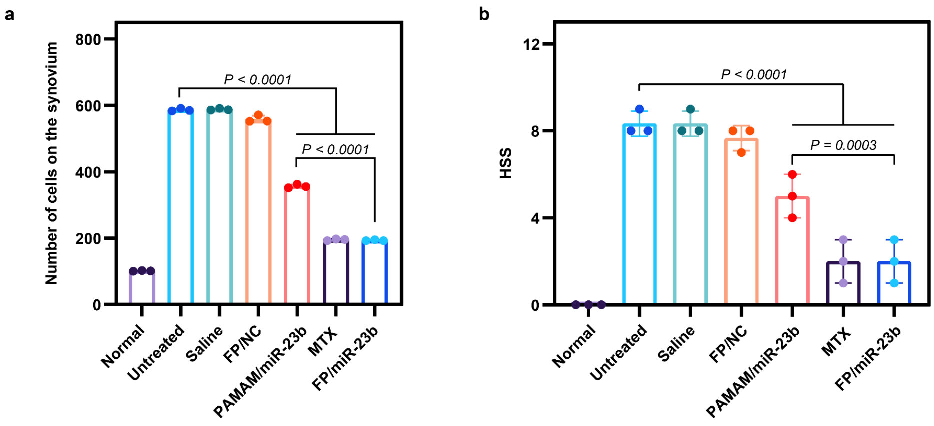

Supplementary Fig. 17. Histological quantification of the arthritic joints in AIA rats. **(a)** Infiltrated cells in the hyperplastic synovium of AIA rats were quantified by Image J software. **(b)** Histological synovitis scores (HSS) of hyperplastic synovium in AIA rats were assessed by microscopic images of H&E staining. Three different regions of the arthritic joints were randomly chosen for analysis. Data are presented as mean value ± SD (n= 3 independent experiments). One-sided statistical analysis is measured by one-way ANOVA with LSD test.

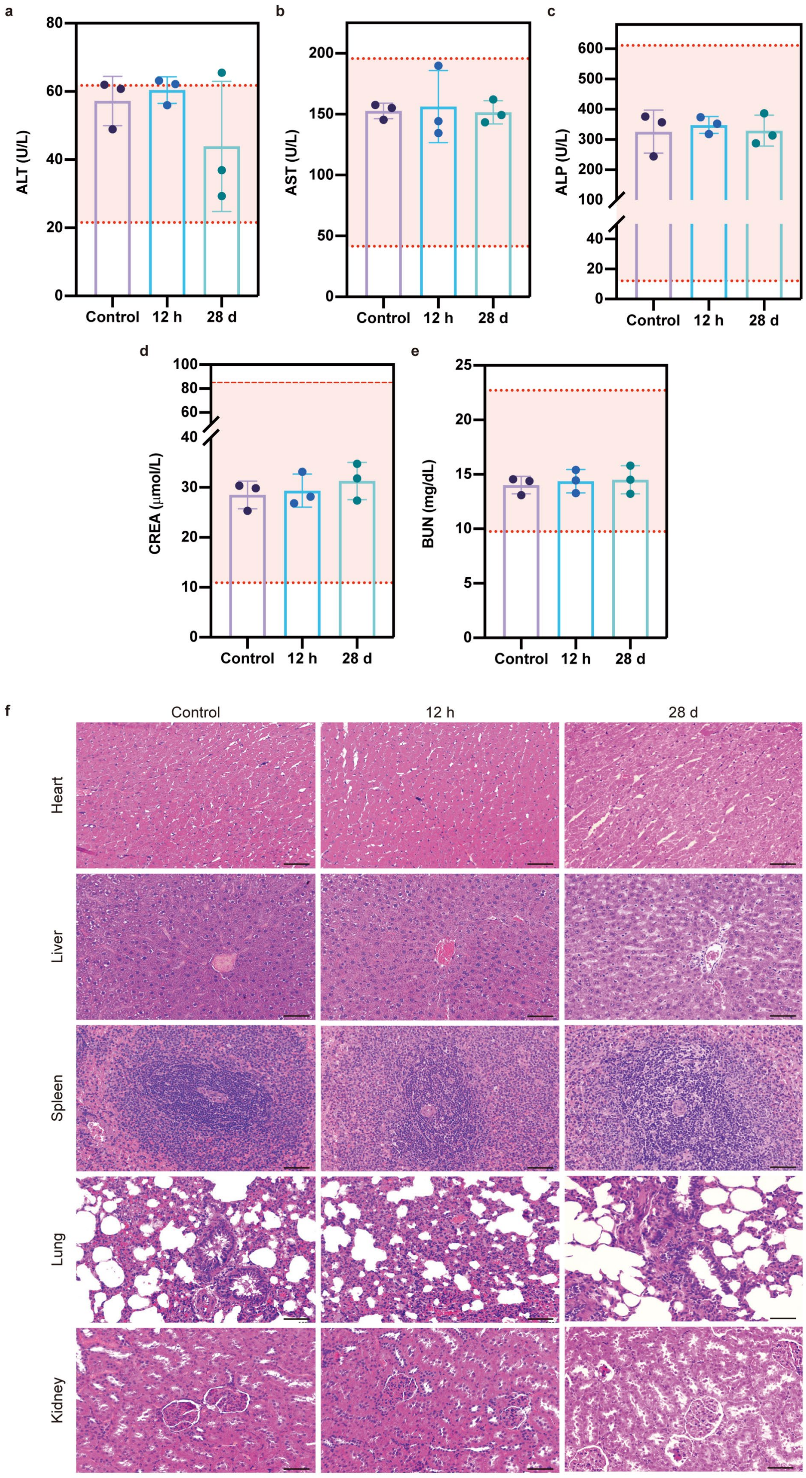

Supplementary Fig. 18. *In vivo* systemic toxicity of nanoparticles in rats. **(a)** Alanine aminotransferase (ALT), **(b)** aspartate aminotransferase (AST), **(c)** alkaline phosphatase (ALP), **(d)** creatine (CREA) and **(e)** blood urea nitrogen (BUN) levels of rats, in which the serum was collected at 12 h and 28 d after the intravenous injection of FP/miR-23b

nanoparticles. The dashed lines present the average range of serum biochemical parameters in the healthy rat. In **a**-**e**, data are presented as mean value ± SD (n= 3 independent animals). **(f)** H&E staining of heart, liver, spleen, lung and kidney of rats, in which tissues were harvested at 12 h and 28 d after the injection of FP/miR-23b nanoparticles. Scale bar: 100 μm. A representative image of three biologically independent samples from each group is shown.

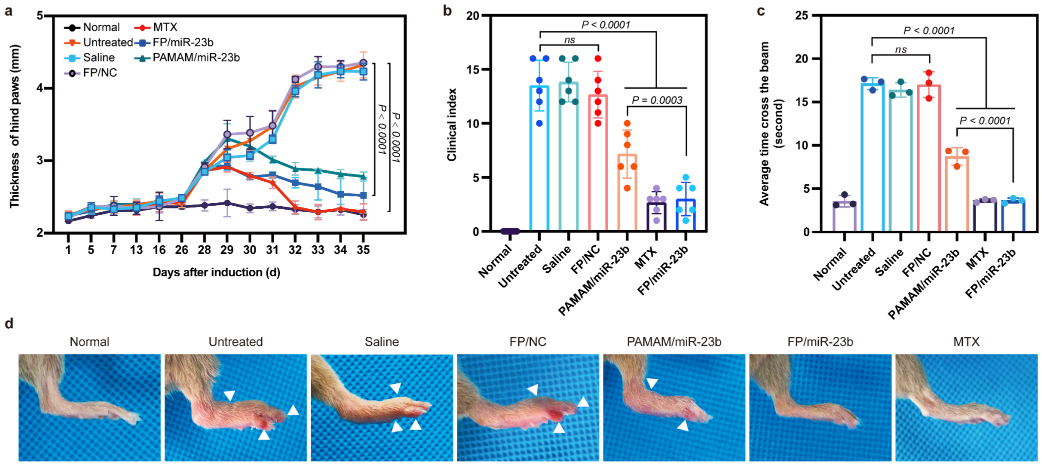

Supplementary Fig. 19. Therapeutic efficacy of FP/miR-23b nanoparticles in CIA rat model in terms of changes in inflamed paws. **(a)** The thickness of hind paws assessed during the treatment. Data are presented as mean value ± SD (n= 3 independent animals). **(b)** The clinical indexes of fore and hind paws assessed at the day 35 post-primary immunization. The clinical indexes were performed on 0-4 scale, where 0 = no signs of swelling or erythema; 1 = slight erythema and/or swelling; 2 = moderate edema; 3 = remarkable edema and limited use of the joint; 4 = excessive edema with joint rigidity. Data are presented as mean value ± SD (n= 6 independent animals). **(c)** The beam walking test assessed at the day 35 post-adjuvant induction, in which the time to cross the 1-m beam was recorded. Data are presented as mean value ± SD (n= 3 independent animals). In **a-c**, one-sided statistical analysis is measured by one-way ANOVA with LSD test. **(d)** The macroscopic observations to assess the severity of soft tissue swelling and bone erosion at the day 35 post primary immunization. White arrows indicate the ankle swelling and redness. A representative image of three independent animals is shown.

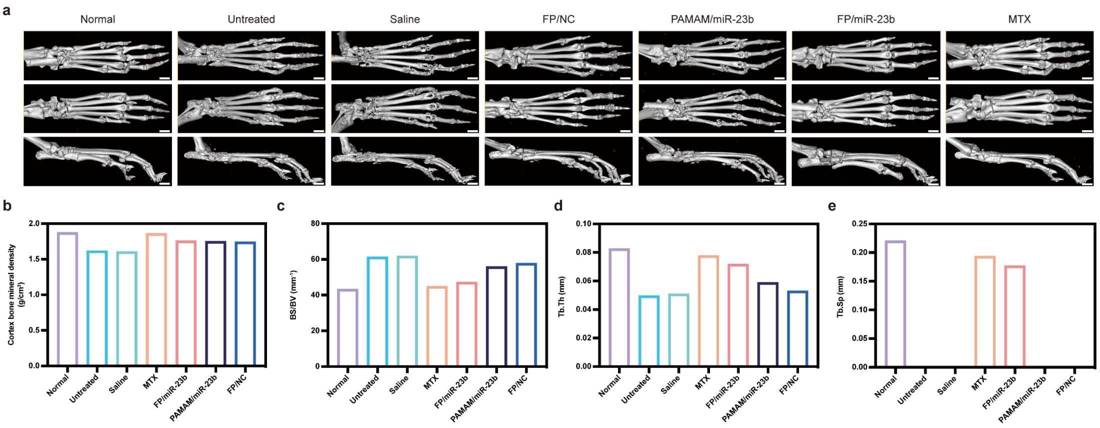

Supplementary Fig. 20. Bone protection of FP/miR-23b nanoparticles in CIA mice. **(a)** The representative micro-CT images of CIA mice after the treatments at the day 33 post primary immunization. **(b-e)** Quantitative micro-CT analysis of **(b)** Cortex bone mineral density (BMD), **(c)** bone surface/bone volume (BS/BV), **(d)** trabecular thickness (Tb. Th) and **(e)** trabecular spacing (Tb. Sp). In **b**-**e**, data are presented as single values from each group.

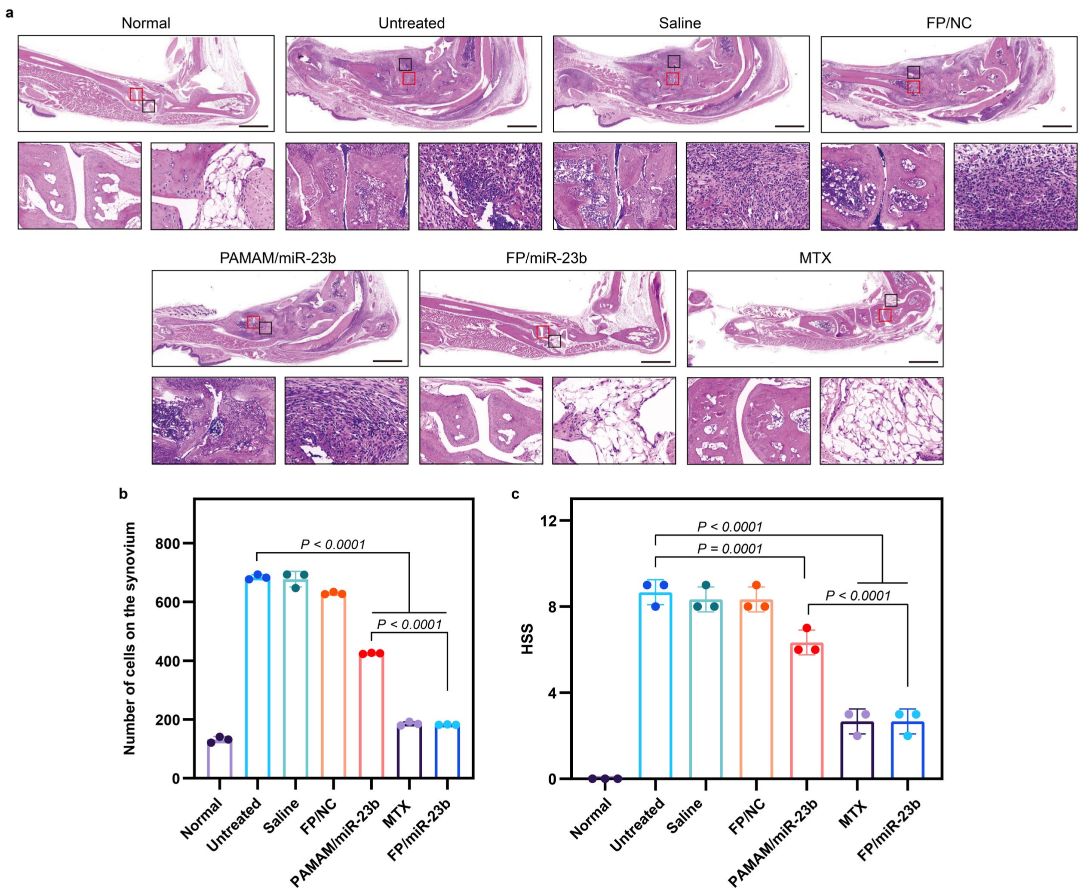

Supplementary Fig. 21. The FP/miR-23b nanoparticles inhibited the cell infiltration in the hyperplastic synovium of CIA mice. **(a)** H&E staining of the arthritic ankles of CIA mice. Scale bar: 1 mm. Red region represents the bone erosion (bottom left) and black region represents the infiltrate of synovial tissues (bottom right). A representative image of three biologically independent samples from each group is shown. **(b)** Infiltrated cells in the hyperplastic synovium of CIA mice were quantified by Image J software. **(c)** Histological synovitis scores (HSS) of hyperplastic synovium in CIA mice were assessed by microscopic images of H&E staining. Three different regions of the arthritic joints were randomly chosen for analysis. In **b** and **c**, data are presented as mean value ± SD (n= 3 independent experiments). One-sided statistical analysis is measured by one-way ANOVA with LSD test.

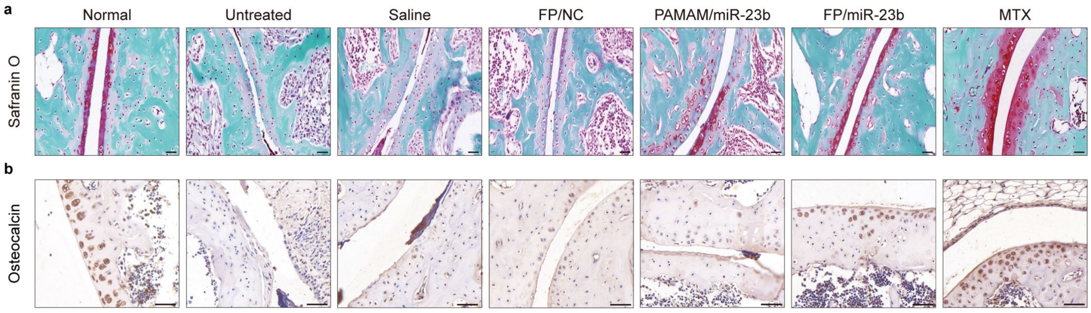

Supplementary Fig. 22. The FP/miR-23b nanoparticles reversed the erosion of articular cartilage in CIA mice. **(a)** Articular cartilages of ankle joints were identified by Safranin O/Fast Green staining. Scale bar: 20 μm **(b)** Expression of the osteocalcin in arthritic joints was detected by the immunochemistry. Scale bar: 20 μm. A representative image of three biologically independent samples from each group is shown in **a** and **b**.

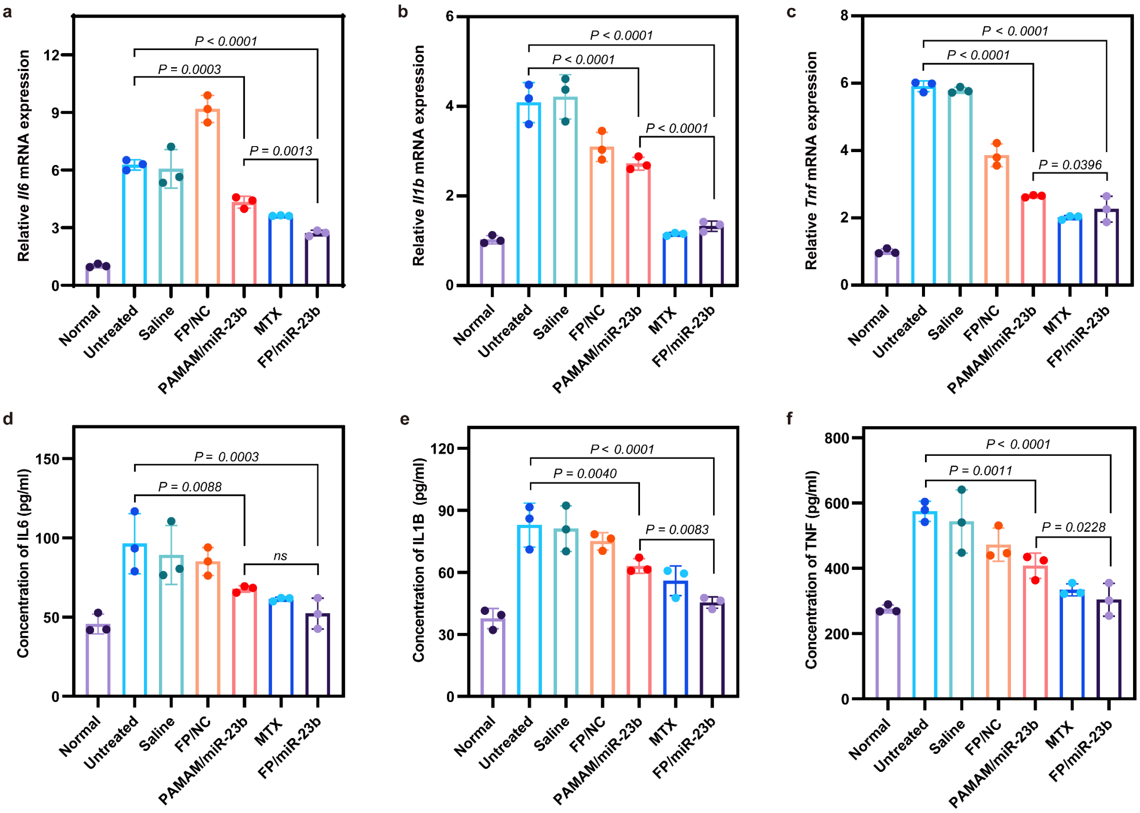

Supplementary Fig. 23. Levels of pro-inflammatory cytokines in CIA mice. **(a-c)** Relative mRNA expression of *Il6* **(a)**, *Il1b* **(b)** and *Tnf* **(c)** in the inflamed synovial tissues determined by qPCR at the day 33 post primary immunization. The expression levels were calculated as the ratio of related cytokines to *Actb*. **(d-f)** Concentrations of pro-inflammatory cytokines in serum (IL6 **(d)**, IL1B **(e)** and TNF **(f)**) determined by ELISA method at the day 33 post primary immunization. Data are presented as mean value ± SD (n= 3 independent animals). One-sided statistical analysis is measured by one-way ANOVA with LSD test.

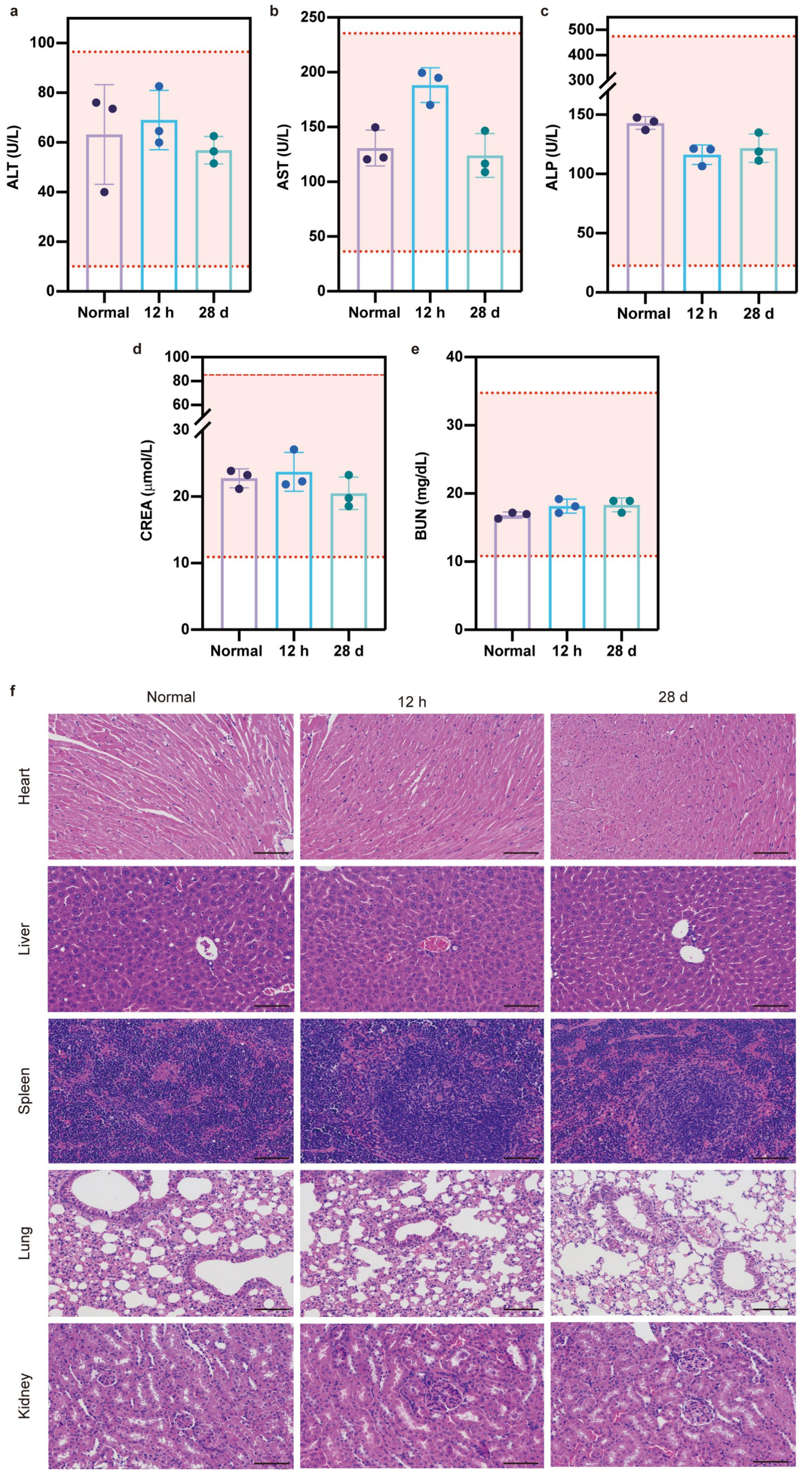

Supplementary Fig. 24. *In vivo* systemic toxicity of nanoparticles in mice. **(a)** Alanine aminotransferase (ALT), **(b)** aspartate aminotransferase (AST), **(c)** alkaline phosphatase (ALP), **(d)** creatine (CREA) and **(e)** blood urea nitrogen (BUN) levels of mice, in which

the serum was collected at 12 h and 28 d after the intravenous injection of FP/miR-23b nanoparticles. The dashed lines present the average range of serum biochemical parameters in the healthy mice. In **a**-**e**, data are presented as mean value ± SD (n= 3 independent animals). **(f)** H&E staining of heart, liver, spleen, lung and kidney of mice, in which tissues were harvested at 12 h and 28 d after the injection of FP/miR-23b nanoparticles. Scale bar:100 μm. A representative image of three biologically independent samples from each group is shown.

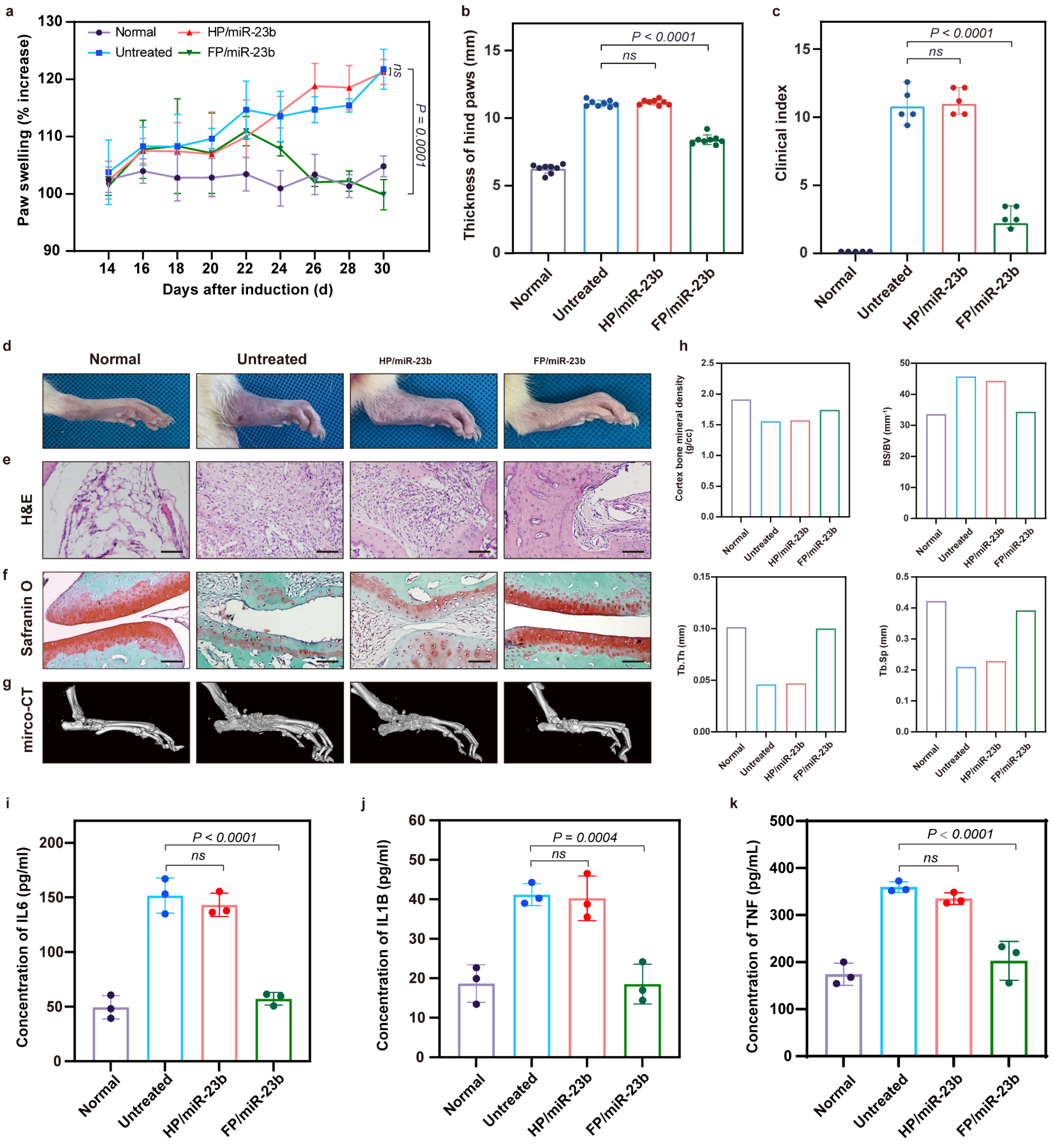

Supplementary Fig. 25. The therapeutic efficacy of HP/miR-23b nanoparticles in AIA rats. **(a)** The increase ratio of hind paw swelling during the treatment. Data are presented as mean value ± SD (n= 3 independent animals) **(b)** The thickness of hind paws assessed at the day 30 post-adjuvant induction. Data are presented as mean value ± SD (n= 8 independent samples). **(c)** The clinical indexes of fore and hind paws assessed at the day 30 post-adjuvant induction. The clinical indexes were performed on 0-4 scale, where 0 = no signs of swelling or erythema; 1 = slight erythema and/or swelling; 2 = moderate

edema; 3 = remarkable edema and limited use of the joint; 4 = excessive edema with joint rigidity. Data are presented as mean value ± SD (n= 5 independent samples). **(d)** The macroscopic observations to assess the severity of soft tissue swelling and bone erosion at day 30 post-adjuvant induction. A representative image of three independent animals from each group is shown. **(e)** Histological change of synovium was analyzed by H&E staining. Scale bar: 100 μm. **(f)** Articular cartilages of ankle joints were identified by Safranin O/Fast Green staining. Scale bar: 100 μm. A representative image of three biologically independent samples from each group is shown in **e** and **f**. **(g)** The representative micro-CT images of arthritis rats after different treatments. **(h)** Quantitative micro-CT analysis of cortex bone mineral density (BMD), bone surface/bone volume (BS/BV), trabecular thickness (Tb. Th) and trabecular spacing (Tb. Sp). Data are presented as single values from each group. **(i-k)** Concentrations of pro-inflammatory cytokines in serum (IL6 **(i)**, IL1B **(j)** and TNF **(k)**) determined by ELISA method. Data are presented as mean value ± SD (n= 3 independent animals). In **a-c**, and **i-k**, one-sided statistical analysis is measured by one-way ANOVA with LSD test.

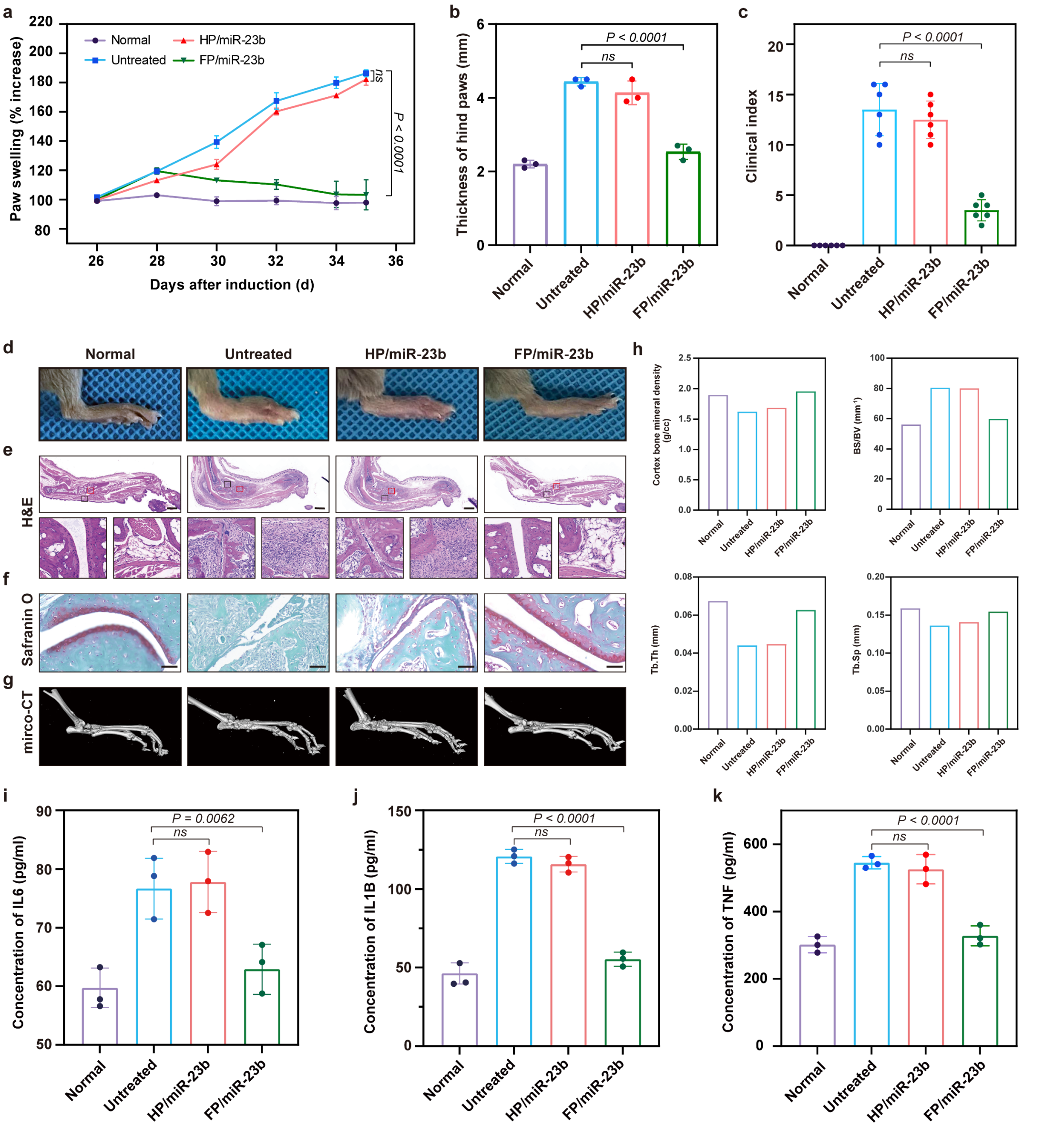

Supplementary Fig. 26. The therapeutic efficacy of HP/miR-23b nanoparticles in CIA mice. **(a)** The increase ratio of hind paw swelling during the treatment. Data are presented as mean value ± SD (n= 3 independent animals). **(b)** The thickness of hind paws assessed at the day 35 post-adjuvant induction. Data are presented as mean value ± SD (n= 3 independent samples). **(c)** The clinical indexes of fore and hind paws assessed at the day 35 post-adjuvant induction. The clinical indexes were performed on 0-4 scale, where 0 = no signs of swelling or erythema; 1 = slight erythema and/or swelling; 2 = moderate edema; 3 = remarkable edema and limited use of the joint; 4 = excessive edema with joint

rigidity. Data are presented as mean value ± SD (n= 6 independent animals). **(d)** The macroscopic observations to assess the severity of soft tissue swelling and bone erosion at the day 35 post primary immunization. A representative image of three independent animals from each group is shown. **(e)** Histological change of synovium was analyzed by H&E staining. Scale bar: 1 mm. **(f)** Articular cartilages of ankle joints were identified by Safranin O/Fast Green staining. Scale bar: 50 μm. A representative image of three biologically independent samples from each group is shown in **e** and **f**. **(g)** The representative micro-CT images of arthritis rats after different treatments. **(h)** Quantitative micro-CT analysis of cortex bone mineral density (BMD), bone surface/bone volume (BS/BV), trabecular thickness (Tb. Th) and trabecular spacing (Tb. Sp). Data are presented as single values from each group. **(i-k)** Concentrations of pro-inflammatory cytokines in serum (IL6 **(i)**, IL1B **(j)** and TNF **(k)**) determined by ELISA method. Data are presented as mean value ± SD (n= 3 independent animals). In **a-c**, and **i-k**, one-sided statistical analysis is measured by one-way ANOVA with LSD test.

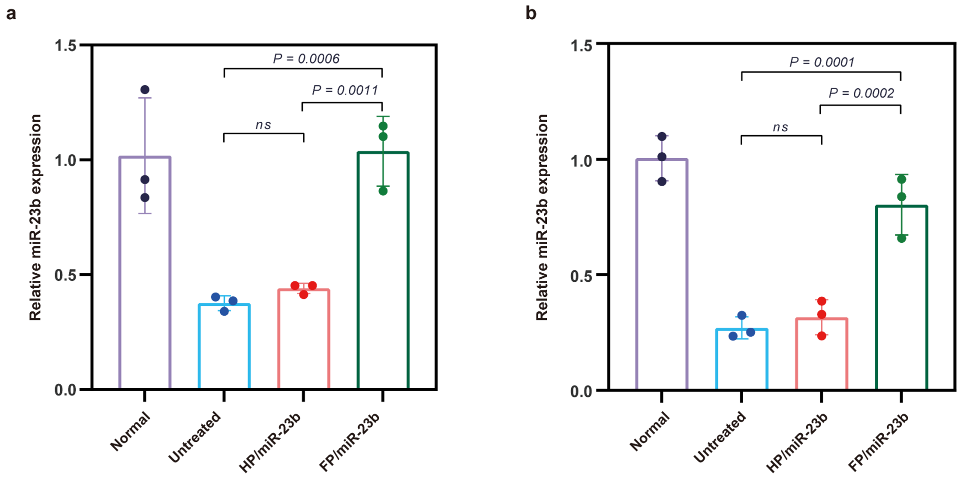

Supplementary Fig. 27. Relative miR-23b expression in the inflamed synovial tissues after the intravenous administration of FP/miR-23b and HP/miR-23b nanoparticles into **(a)** AIA rats and **(b)** CIA mice at the end of the treatment. Data are presented as mean value ± SD (n= 3 independent samples). One-sided statistical analysis is measured by one-way ANOVA with LSD test.

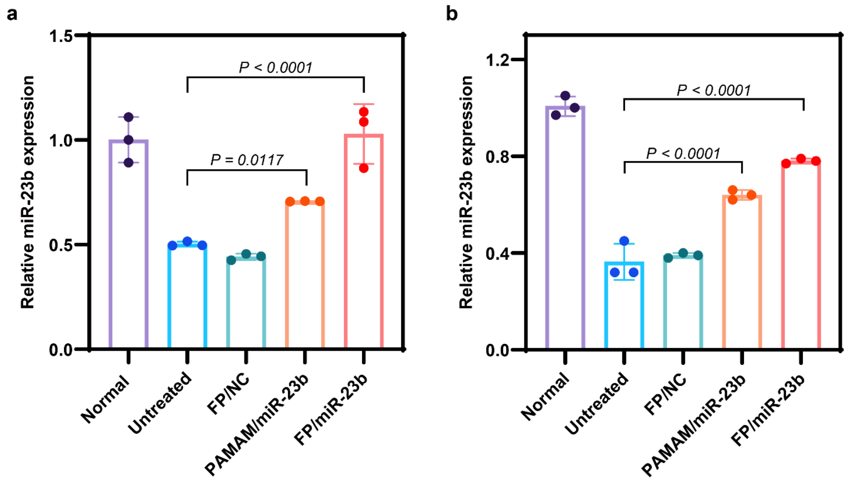

Supplementary Fig. 28. Relative miR-23b expression in the inflamed synovial tissues after the intravenous administration of FP/miR-23b nanoparticles into **(a)** AIA rats and **(b)** CIA mice at the end of the treatment. Data are presented as mean value ± SD (n= 3 independent samples). One-sided statistical analysis is measured by one-way ANOVA with LSD test.

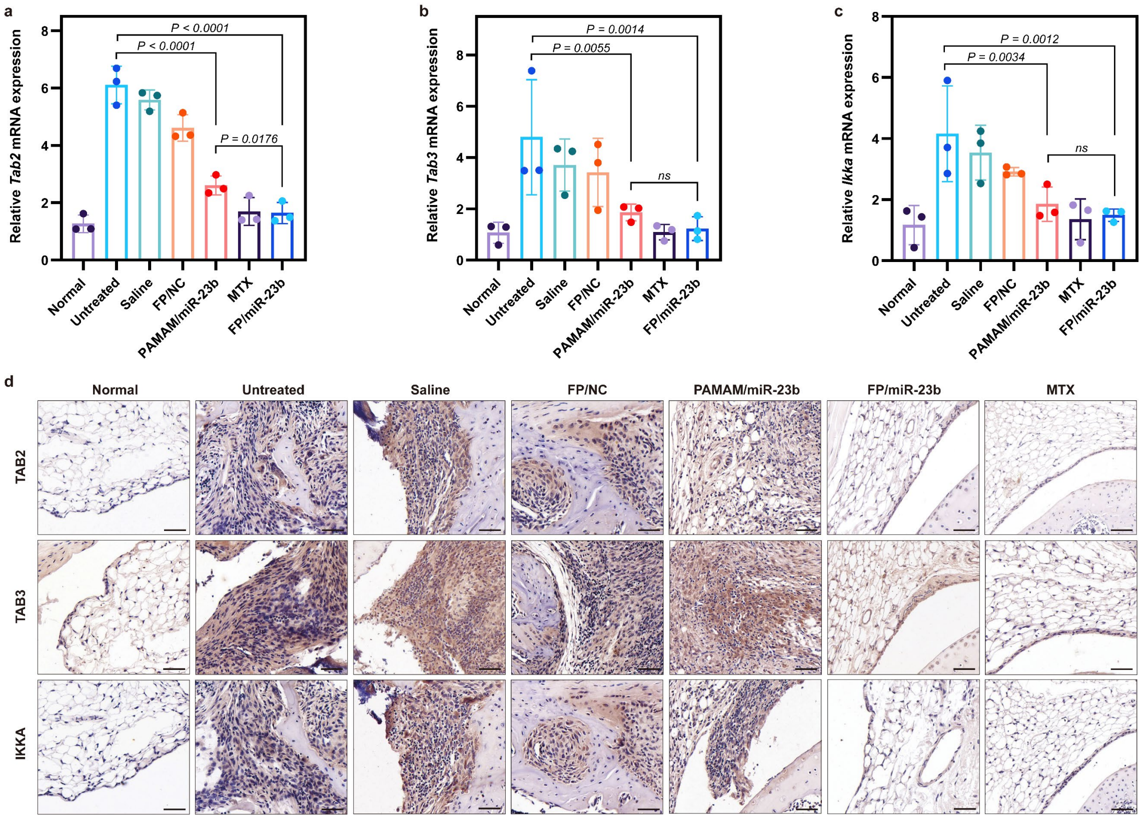

Supplementary Fig. 29. The FP/miR-23b nanoparticles regulated the NF-κB signaling pathway in the synovium of CIA mice. **(a-c)** The mRNA expression of **(a)** *Tab2*, **(b)** *Tab3* and **(c)** *Ikka* in the arthritic joints of CIA mice were determined by qPCR. Data are presented as mean value ± SD (n= 3 independent samples). One-sided statistical analysis is measured by one-way ANOVA with LSD test. **(d)** Levels of TAB2, TAB3 and IKKA in the arthritic joints of CIA mice were determined by immunochemistry. Scale bar: 50 μm. A representative image of three biologically independent samples from each group is shown.

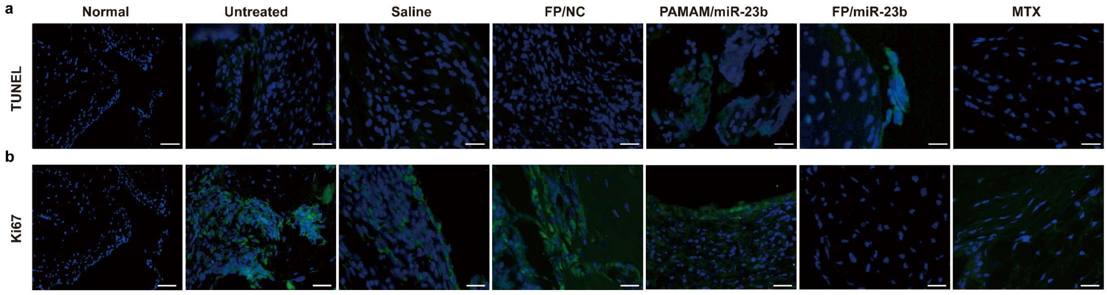

Supplementary Fig. 30. The anti-proliferative effect of FP/miR-23b nanoparticles in the synovial tissues of CIA mice by TUNEL **(a)** and Ki67 **(b)** immunofluorescence staining. Nuclei, blue (DAPI); TUNEL or Ki67, green. Scale bar: 20 μm. A representative image of three biologically independent samples from each group is shown in **a** and **b**.

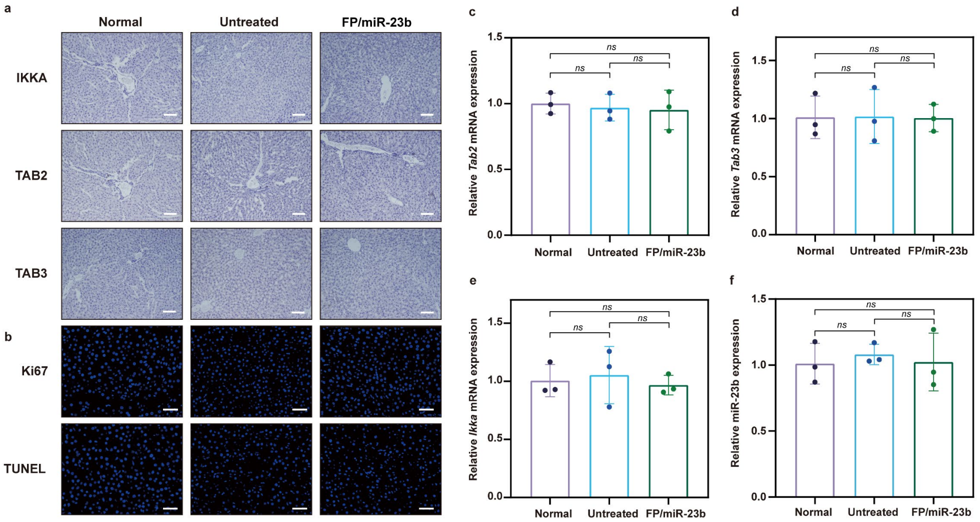

Supplementary Fig. 31. The FP/miR-23b nanoparticles barely regulated the NF-κB signaling pathway and apoptotic process in the liver of AIA rats. **(a)** Levels of TAB2, TAB3 and IKKA in the liver of AIA rats were determined by immunochemistry. Scale bar: 200 µm. **(b)** The anti-proliferative effect of FP/miR-23b nanoparticles in the liver of AIA rats was evaluated by TUNEL and Ki67 immunofluorescence staining. Nuclei, blue (DAPI); TUNEL or Ki67, green. Scale bar: 50 µm. A representative image of three biologically independent samples from each group is shown in **a** and **b**. **(c-f)** The hepatic expression levels of **(c)** *Tab2*, **(d)** *Tab3*, **(e)** *Ikka* and **(f)** miR-23b in AIA rats were determined by qPCR at the end of treatment. Data are presented as mean value ± SD (n= 3 independent samples). One-sided statistical analysis is measured by one-way ANOVA with LSD test.

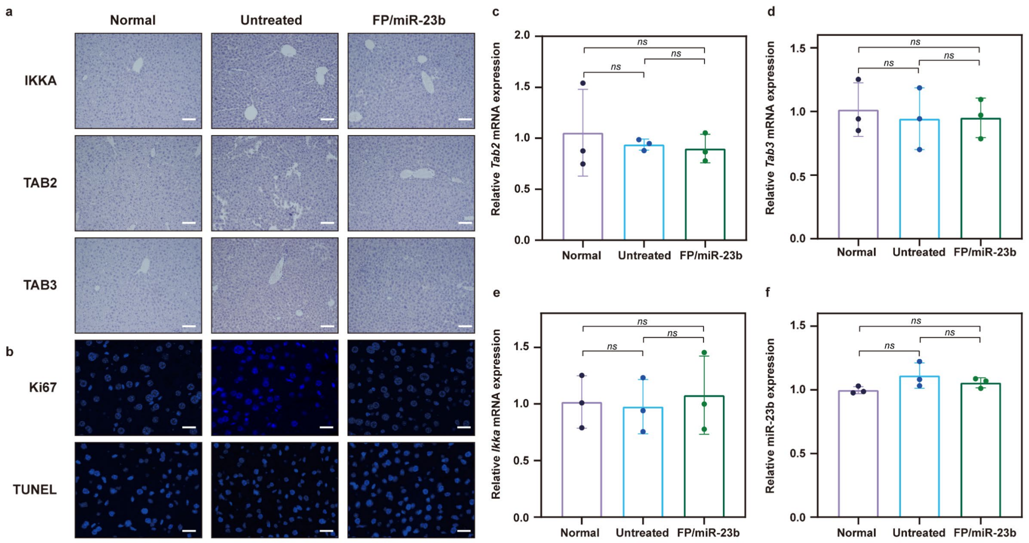

Supplementary Fig. 32. The FP/miR-23b nanoparticles barely regulated the NF-κB signaling pathway and apoptotic process in the liver of CIA mice. **(a)** Levels of TAB2, TAB3 and IKKA in the liver of CIA mice were determined by immunochemistry. Scale bar: 200 μm. **(b)** The anti-proliferative effect of FP/miR-23b nanoparticles in the liver of CIA mice was evaluated by TUNEL and Ki67 immunofluorescence staining. Nuclei, blue (DAPI); TUNEL or Ki67, green. Scale bar: 20 μm. A representative image of three biologically independent samples from each group is shown in **a** and **b**. **(c-f)** The hepatic expression levels of **(c)** *Tab2*, **(d)** *Tab3*, **(e)** *Ikka* and **(f)** miR-23b in CIA mice were determined by qPCR at the end of treatment. Data are presented as mean value ± SD (n= 3 independent samples). One-sided statistical analysis is measured by one-way ANOVA with LSD test.

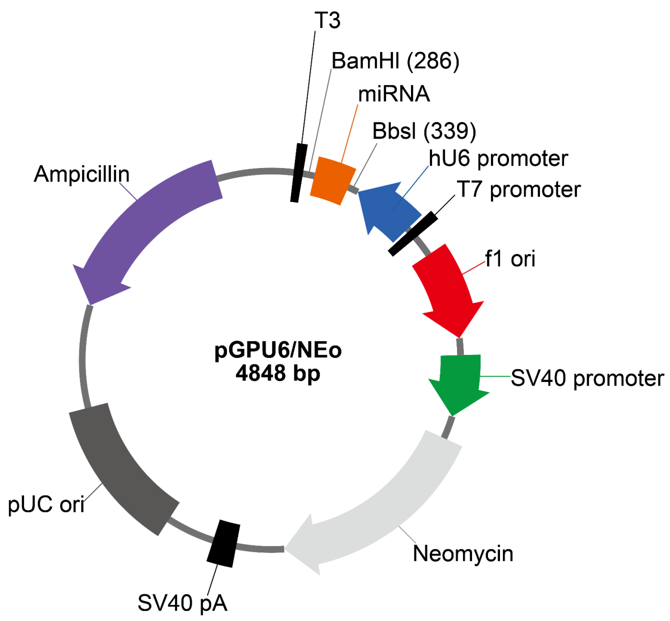

Supplementary Fig. 33. The plasmid map of pGPU6/Neo expressing miR-23b (5'-ATCACATTGCCAGGGATTACC-3') or negative control miRNA (5'-TTCTCCGAACGTGTCACGT-3').

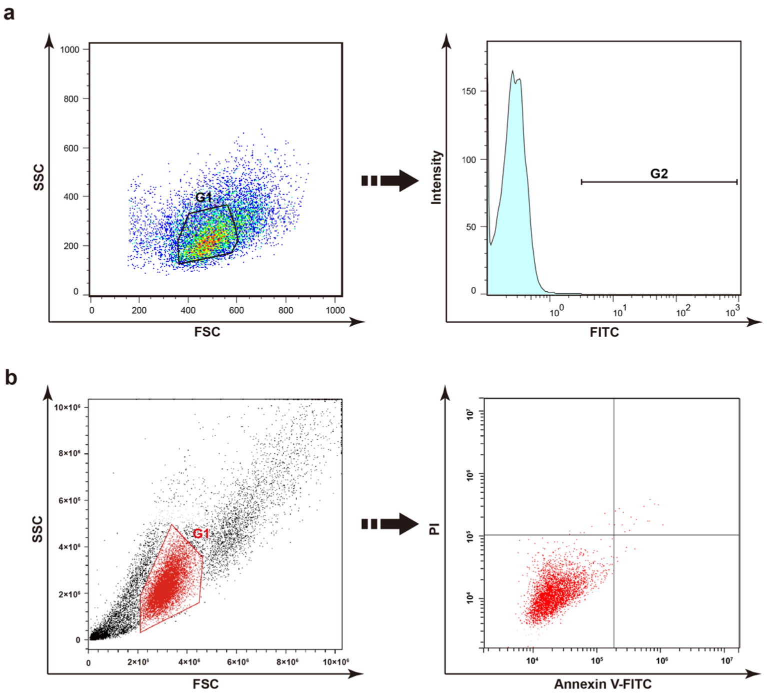

Supplementary Fig. 34. Gating strategies used for endocytosis and apoptosis. **(a)** Gating strategy for the endocytosis of FP/miR-23b nanoparticles by RAW264.7 cells. The cells demonstrating the normal morphology were gated first (left panel, G1) and their FITC fluorescence in G1 was analyzed (right panel, G2). **(b)** Gating strategy for the apoptosis of FP/miR-23b nanoparticles in BMDMs. The cells demonstrating the normal morphology were gated first (left panel, G1) and the fluorescent distribution of PI and FITC in G1 were then analyzed to evaluate the stage of cell apoptosis.

Supplementary table 1 The primers used in the mRNA/miRNA expression analysis experiment.

Primer	Sequence (5'-3')
Mouse *Tnf* forward primer	CCACCACGCTCTTCTGTCTAC
Mouse *Tnf* reverse primer	GCCATTTGGGAACTTCTCATC
Mouse *Il1b* forward primer	TTGACGGACCCCAAAAGATG
Mouse *Il1b* reverse primer	AGAAGGTGCTCATGTCCTCA
Mouse *Il6* forward primer	AACGATGATGCACTTGCAGA
Mouse *Il6* reverse primer	GAGCATTGGAAATTGGGGTA
Mouse *Actb* forward primer	AGTGTGACGTTGACATCCGT
Mouse *Actb* reverse primer	GCAGCTCAGTAACAGTCCGC
Mouse *Tab2* forward primer	AGAAGTGCCTGAAGTTGTTGTATC
Mouse *Tab2* reverse primer	TGTTAAAGTCCTGCTTCCATTC
Mouse *Tab3* forward primer	ATCAGCGAGCAAGAATGGAGA
Mouse *Tab3* reverse primer	GTTCGTGCTTCTCAATCTTGTCAT
Mouse *Ikka* forward primer	TGAGCGTGAAACAGGAATAAATAC
Mouse *Ikka* forward primer	GCAAATGGTCCTTCATATACAGTC
Rat *Tnf* forward primer	GTCGTAGCAAACCACCAAGC
Rat *Tnf* reverse primer	CTCCTGGTATGAAATGGCAAA
Rat *Il1b* forward primer	TGACCCATGTGAGCTGAAAG
Rat *Il1b* reverse primer	AGGGATTTTGTCGTTGCTTG
Rat *Actb* forward primer	CGAGTACAACCTTCTTGCAGC
Rat *Actb* reverse primer	ACCCATACCCACCATCACAC
Rat *Il6* forward primer	TTCACAGAGGATACCACCCACA

Rat *Il6* reverse primer	GCATCATCGCTGTTCATACAATC
Rat *Tab2* forward primer	CTGAAGTGCCTGAAGTTGTTGT
Rat *Tab2* reverse primer	GAGAAGTCATGTGATTGCGTAGA
Rat *Tab3* forward primer	CACGCCTGAGGAAATGACAAG
Rat *Tab3* reverse primer	TAGATGGCTTCGGTGGTACAAC
Rat *Ikka* forward primer	ACAGACCCACCGATCACTCCTA
Rat *Ikka* forward primer	AGCCCAACAACTTGCTCAGGT
Mouse-RT-miR-23b	GTCGTATCCAGTGCGTGTCGTGGAGTC GGCAATTGCACTGGATACGACGGTAAT
Mouse-miR-23b forward primer	GCATCACATTGCCAGGG
Mouse-miR-23b reserve primer	CAGTGCGTGTCGTGGAGT
Rat-RT-miR-23b	CTCAACTGGTGTCGTGGAGTCGGCAATTCAG TTGAGGGTAATC
Rat-miR-23b forward primer	ACACTCCAGCTGGGATCACATTGCCAGG
Rat-miR-23b reserve primer	TGGTGTCGTGGAGTCG
U6 forward primer	CTCGCTTCGGCAGCACA
U6 reserve primer	AACGCTTCACGAATTTGCGT

Supplementary table 2 Histological synovitis scores (HSS) of inflamed tissues

Feature	Score
A. Hyperplasia or enlargement of synovial lining cell layer	
1. Absent	
2. Slight enlargement (two to three cell layers). Giant cells are very rare	0
3. Moderate enlargement (four to five cell layers). Some giant cells or	1
lymphocytes	2
4. Strong enlargement (more than six cell layers). Giant cells and	3
lymphocytes are frequent	
B. Inflammatory infiltration	
1. Absent	
2. Slight inflammatory infiltration (diffusely located single cells and	0
small perivascular aggregates of lymphocytes and/or plasma cells)	1
3. Moderate inflammatory infiltration (perivascular and/or superficial	
lymphatic aggregates, and small sized lymphatic follicles without germinal	2
center may be observed)	
4. Strong inflammatory infiltration (lymphatic follicles with germinal	3
center and/or confluent subsynovial lymphatic infiltration)	
C. Activation of synovial stroma/pannus formation	0
1. Absent	1
2. Slight synovial stroma activation (low cellularity with slight edema,	
slight fibrosis with some fibroblast, no giant cells)	2

3. Moderate synovial stroma activation (moderate cellularity with a moderate density of fibroblasts, endothelial cells, and giant cells may be detected)<br>4. Strong synovial stroma activation (high cellularity with dense distribution of fibroblasts and endothelial cells, and giant cells are abundant)	3

Supplementary method

The ninhydrin assays. The number of the fluorinated groups on PAMAM was measured by the ninhydrin assay. Briefly, 15 mg hydrindantin and 85 mg ninhydrin were dissolved 10 ml ethylene glycol-monomethyl ether. The mixture (100 μl) was added into 100 μl of sodium acetate buffer (0.2 M, pH 5.4). The solution was mixed with a series concentration of PAMAM and fixed the final volume of 300 μl using distilled water. The mixture was then heated to 100 °C for 10 min and then cooled to room temperature. Afterwards, 60% (v/v) ethanol/water solution (300 μl) was added into each sample, and the absorbance was detected at 570 nm. The calibration curve was established according to the absorbance and PAMAM concentrations.

Therapeutic efficacy of HP/miR-23b nanoparticles in AIA rats. Briefly, each Lewis rat (aged 6 weeks) was immunized subcutaneously with 100 μl complete Freund's adjuvant containing 5 mg/ml *Mycobacterium tuberculosis* at the base of the tail. After post-induction for ~12 days, rats appeared the symptoms of joint inflammation and pain, with the swelling and redness of paws. Once the signs of inflammation were evident, twenty-four rats were randomly assigned to four groups (n = 6) as follows: Group 1 was healthy Lewis rats; Group 2 with arthritis received no therapeutic treatment was served as control; Groups 3 and 4 with arthritis were received intravenous injection of FP/miR-23b (N/P ratio of 4.0) and HP/miR-23b (N/P ratio of 4.0), respectively. The AIA rats were intravenously injected with nanoparticles on day 16 and 20 after the adjuvant induction (miR-23b dose of 0.5 mg/kg body weight). The clinical parameters were monitored during the experiment, including the clinical index, mobility, the thickness of hind paws.

Therapeutic efficacy of HP/miR-23b nanoparticles in CIA mice. Briefly, each DBA1/J mouse (aged 8 weeks) were subcutaneously immunized by 100 μg type II bovine

collagen (2 mg/ml) emulsified in 50 μl CFA containing 2 mg/ml *Mycobacterium tuberculosis* at the base of the tail. On day 21, a booster injection with the collagen and IFA emulsion was administered into the mice to induce the arthritis. Twenty-four mice were divided into four groups (n = 6 per group) as described above. The CIA mice were intravenously administered with the nanoparticles five times (once every other day) after 24 days post primary immunization. During the treatment, the thickness of hind paws and the clinical indexes were monitored.

Immunochemical analysis of liver tissues. The embedded liver tissues were sectioned into 10 μm-thickness slices and then applied for the antigen unmasking in EDTA solution (Daixuan, Shanghai, China) for 12 min under the microwave. The hydrogen peroxide blocking was conducted using 3% (v/v) hydrogen peroxide solution for 25 min in the dark. Afterwards, the slides were blocked with 3% (w/v) BSA solution diluted in PBS buffer for 30 min and then incubated with individual primary antibody at 4 °C overnight. The immunochemical analysis was conducted by applying HRP-conjugated secondary antibody at room temperature for 1 h, followed by the visualization of diaminobenzidine (DAB) substrate and hematoxylin. Finally, the sectioned tissues were rehydrated in ethanol and xylene, and mounted by neutral balsam. The slides were observed by Olympus DP70 microscopy (Tokyo, Japan).

Anti-proliferative analysis of liver tissues. After the treatment of the arthritic animals, the liver tissues were dissected for the section. The parafilm embedded livers were sectioned for TUNEL and anti-Ki67 staining, and the nucleus was stained by DAPI. The slides were observed by Olympus DP70 microscopy (Tokyo, Japan).
