# Supplementary material for: Fluorinated polyamidoamine dendrimer-mediated miR-23b delivery for the treatment of experimental rheumatoid arthritis in rats
Source: Nat Commun. 2023 Feb 20;14:944. doi: 10.1038/s41467-023-36625-7 (PMC9941585; doi:10.1038/s41467-023-36625-7)
Supplement: Supplementary file 3 — Description of Additional Supplementary Files [file 41467_2023_36625_MOESM3_ESM.pdf]

Description of Additional Supplementary Files

File name: Supplementary Movie 1

Description: The beam walking test of AIA rats received with different treatments including saline, FP/NC nanoparticles, PAMAM/miR-23b nanoparticles, FP/miR-23b nanoparticles and methotrexate (MTX).
