# Supplementary material for: Fluorinated polyamidoamine dendrimer-mediated miR-23b delivery for the treatment of experimental rheumatoid arthritis in rats
Source: Nat Commun. 2023 Feb 20;14:944. doi: 10.1038/s41467-023-36625-7 (PMC9941585; doi:10.1038/s41467-023-36625-7)
Supplement: Supplementary file 5 — Reporting Summary [file 41467_2023_36625_MOESM5_ESM.pdf]

nature portfolio

Corresponding author(s): Quanshun Li

Last updated by author(s): Jan 16, 2023

Reporting Summary

Nature Portfolio wishes to improve the reproducibility of the work that we publish. This form provides structure for consistency and transparency in reporting. For further information on Nature Portfolio policies, see our Editorial Policies and the Editorial Policy Checklist.

Statistics

For all statistical analyses, confirm that the following items are present in the figure legend, table legend, main text, or Methods section.

n/a	Confirmed	
☐	☒	The exact sample size (*n*) for each experimental group/condition, given as a discrete number and unit of measurement
☐	☒	A statement on whether measurements were taken from distinct samples or whether the same sample was measured repeatedly
☐	☒	The statistical test(s) used AND whether they are one- or two-sided<br>*Only common tests should be described solely by name; describe more complex techniques in the Methods section.*
☒	☐	A description of all covariates tested
☐	☒	A description of any assumptions or corrections, such as tests of normality and adjustment for multiple comparisons
☐	☒	A full description of the statistical parameters including central tendency (e.g. means) or other basic estimates (e.g. regression coefficient) AND variation (e.g. standard deviation) or associated estimates of uncertainty (e.g. confidence intervals)
☐	☒	For null hypothesis testing, the test statistic (e.g. *F*, *t*, *r*) with confidence intervals, effect sizes, degrees of freedom and *P* value noted<br>*Give P values as exact values whenever suitable.*
☒	☐	For Bayesian analysis, information on the choice of priors and Markov chain Monte Carlo settings
☒	☐	For hierarchical and complex designs, identification of the appropriate level for tests and full reporting of outcomes
☒	☐	Estimates of effect sizes (e.g. Cohen's *d*, Pearson's *r*), indicating how they were calculated

Our web collection on statistics for biologists contains articles on many of the points above.

Software and code

Policy information about availability of computer code

Data collection	CellQUEST Pro v6.0;<br>Malvern Zetasizer Software 7.11;<br>ABI 7500 Software v2.0.6.
Data analysis	Flowjo v10.0;<br>CytExpert 2.3;<br>Graphpad Prism 8.0;<br>Materialize mimics 19.0.<br>Image J software v1.5j8

For manuscripts utilizing custom algorithms or software that are central to the research but not yet described in published literature, software must be made available to editors and reviewers. We strongly encourage code deposition in a community repository (e.g. GitHub). See the Nature Portfolio guidelines for submitting code & software for further information.

Data

Policy information about availability of data

All manuscripts must include a data availability statement. This statement should provide the following information, where applicable:

- Accession codes, unique identifiers, or web links for publicly available datasets
- A description of any restrictions on data availability
- For clinical datasets or third party data, please ensure that the statement adheres to our policy

All data are available in the Article, Supplementary Information files, or from the corresponding author upon reasonable request. The source data underlying Figs. 1b, 2d, e, 3a, d, 4, 5a-c, 6b-c, 8, 9b-d, 10a-c and Supplementary Figs. 2, 5, 6, 7, 9b,10, 12a, e, 13, 14a, 15a-c, 16, 17, 18a-e, 19a-c, 20b-e, 21b, c, 23, 24a-e, 25a-c, h-k, 26a-c, h-k, 27, 28, 29a-c, 31c-f, and 32c-f are provided as a Source Data file. Source data are provided with this paper.

Human research participants

Policy information about studies involving human research participants and Sex and Gender in Research.

Reporting on sex and gender	N/A
Population characteristics	N/A
Recruitment	N/A
Ethics oversight	N/A

Note that full information on the approval of the study protocol must also be provided in the manuscript.

Field-specific reporting

Please select the one below that is the best fit for your research. If you are not sure, read the appropriate sections before making your selection.

☒ Life sciences ☐ Behavioural & social sciences ☐ Ecological, evolutionary & environmental sciences

For a reference copy of the document with all sections, see nature.com/documents/nr-reporting-summary-flat.pdf

Life sciences study design

All studies must disclose on these points even when the disclosure is negative.

Sample size	No sample size calculation was performed. Sample size was determined to be sufficient based on the magnitude and consistency of measurable differences between groups. In addition, the sample sizes of this study were determined on the basis of similar published studies. In vitro experiments, the sample size for each group was 3. Because the reduction of experimental animals is an important principle for animal experiments, the in vivo therapeutic efficacy studies were conducted with 6 animals per group, which also reached statistical significance ($p<0.05$) between experimental groups. Meanwhile, sample sizes for the in vivo experiments are similar to those generally employed and accepted in the field (Nat. Commun. 8, 146 (2017); Nat. Med. 18, 1077 (2012)) and are adequate to support our conclusions with statistical significance.
Data exclusions	None.
Replication	Experimental results were reproducible. Replicate numbers are indicated in the figure legends.
Randomization	Samples were randomly allocated to corresponding experimental groups. Cells were cultured and maintained in the same environment and randomly allocated to each group. For in vivo study, experimental animals were randomly allocated into each groups once the signs of arthritic inflammation were evident.
Blinding	Investigator was blinded to the different groups at the time of measurement.

Reporting for specific materials, systems and methods

We require information from authors about some types of materials, experimental systems and methods used in many studies. Here, indicate whether each material, system or method listed is relevant to your study. If you are not sure if a list item applies to your research, read the appropriate section before selecting a response.

Materials & experimental systems

n/a	Involved in the study
☐	☒ Antibodies
☐	☒ Eukaryotic cell lines
☒	☐ Palaeontology and archaeology
☐	☒ Animals and other organisms
☒	☐ Clinical data
☒	☐ Dual use research of concern

Methods

n/a	Involved in the study
☒	☐ ChIP-seq
☐	☒ Flow cytometry
☒	☐ MRI-based neuroimaging

Antibodies

Antibodies used	Rb mAb to TAB2 (Cell Signaling Technology, #3745, clone C88H10, lot 1, dilution 1:1000);<br>Rb mAb to IKKA (Cell Signaling Technology, #2682S, NA, lot 5, dilution 1:1000);<br>Rb mAb to TAB3 (Abcam, #ab124723, clone EPR5965, lot GR83293-9, dilution 1:5000);<br>Rb mAb to ACTB (Abcam, #ab227387, NA, lot GR3263411-3, dilution 1:5000);<br>Rb mAb to IKKA (Abcam, #ab32041, clone Y463, lot GR117080-29, GR3368230-15, dilution 1:1000);<br>Rat mAb to F4/80 (Abcam, #ab16911, clone BM8, lot GR3297595-4, dilution 1:50);<br>Rb mAb to Ki67 (Abcam, # ab16667, clone SP6, lot GR3375640-34, dilution 1:200);<br>Goat Anti-Rabbit IgG H&L (HRP) (Abcam, #ab6721, NA, lot GR172025-4, GR297013-3, dilution 1:5000);<br>Rb mAb to TAB2 (Novus, NBP2-68833, NA, lot R92304, dilution 1:1000);<br>Rb mAb to Cadherin 11 (Novus, NBP2-15661, NA, lot 44034, dilution 1:250);<br>Rb mAb to TAB3 (Invitrogen, PA5-116885, NA, lot 3364EA11, 35E8BA35, dilution 1:1000);<br>Rb mAb to Osteocalcin (Proteintech, 23418-1-AP, NA, lot GR3313195-53, dilution 1:100);<br>Peroxidase AffiniPure Goat Anti-Rabbit IgG (H+L) (Jacksonimmunoresearch, 111-035-045, NA, lot 00000130634, dilution 1:500);<br>Alexa Fluor 488 goat anti-rabbit IgG secondary antibody (Jacksonimmunoresearch, 111-545-003, NA, lot 000000101724, dilution 1:500).
Validation	All commercially available antibodies are validated by the manufacturers as indicated on the respective manufacturer's website. Manufacturer's website containing validation data for the commercially available antibodies and citations for the custom-made antibodies are listed below:<br>1.TAB2: Mouse; WB (https://www.cellsignal.com/products/primary-antibodies/tab2-c88h10-rabbit-mab/3745);<br>2. IKKA: Mouse; WB (https://www.cellsignal.com/products/primary-antibodies/ikka-antibody/2682?site-search-type=Products&N=4294956287&Ntt=2682s&fromPage=<br>plp& requestid=6946852);<br>3. TAB3: Mouse; WB (https://www.abcam.cn/tab3-antibody-epr5965-ab124723.html);<br>4. ACTB: Mouse; WB (https://www.abcam.cn/beta-actin-antibody-ab227387.html);<br>5. Goat Anti-Rabbit IgG H&L (HRP): WB (https://www.abcam.cn/goat-rabbit-igg-hl-hrp-ab6721.html);<br>6. IKKα: Mouse, rat; IHC (https://www.abcam.cn/ikk-alpha-antibody-y463-ab32041.html);<br>7. F4/80: Mouse; IHC (https://www.abcam.cn/f480-antibody-bm8-ab16911.html);<br>8. Ki67: Mouse, rat; IHC (https://www.abcam.cn/ki67-antibody-sp6-ab16667.html);<br>9. TAB2: Mouse, rat; IHC (https://www.novusbio.com/products/tab2-antibody_nbp2-68833);<br>10. Cadherin 11: Mouse; IHC (https://www.novusbio.com/products/cadherin-11-antibody_nbp2-15661);<br>11. TAB3: Mouse, rat; IHC (https://www.thermofisher.cn/cn/zh/antibody/product/TAB3-Antibody-Polyclonal/PA5-116885);<br>12. Osteocalcin: Mouse, rat; IHC (https://www.ptgcn.com/products/Osteocalcin-Antibody-23418-1-AP.htm);<br>13. Peroxidase AffiniPure Goat Anti-Rabbit IgG (H+L): IHC (https://www.jacksonimmuno.com/catalog/products/111-035-045);<br>14. Alexa Fluor 488 goat anti-rabbit IgG secondary antibody: Mouse; IHC (https://www.jacksonimmuno.com/catalog/<br>products/111-545-003).

Eukaryotic cell lines

Policy information about cell lines and Sex and Gender in Research

Cell line source(s)	RAW264.7, HeLa, NIH3T3 cells were originally obtained from ATCC, in which RAW264.7 cells and NIH3T3 cells were kindly provided by Fu lab and Chen lab in Jilin University, respectively. Murine bone marrow-derived cells were isolated from C57/BL mice (aged 4 weeks) and cultured with DMEM containing 50 ng/ml mM-CSF.
Authentication	None of the cell lines used were authenticated.
Mycoplasma contamination	Cell lines were not tested for mycoplasma contamination.
Commonly misidentified lines<br>(See ICLAC register)	No commonly misidentified cell lines were used in the study.

Animals and other research organisms

Policy information about studies involving animals; ARRIVE guidelines recommended for reporting animal research, and Sex and Gender in Research

Laboratory animals	Male Lewis rats (200 ± 20 g, 5 weeks old) and male C57BL/6J mice (15 ± 2 g, 4 weeks old) were purchased from Beijing Vital River Laboratory Animal Technology Co., Ltd. (Beijing, China). Male DBA1/J mice (15 ± 2 g, 7 weeks old) were obtained from Shanghai SLAC Laboratory Animal Technology Co., Ltd. (Shanghai, China). All animals were housed in specific pathogen-free conditions at a temperature of 18 - 23°C with 40 - 60% humidity under standard 12-h light/12-h dark cycle and allowed free access to food and water.
Wild animals	This study did not involve wild animals.
Reporting on sex	Only male animals were considered in this study, and sex was not considered in study design. Previous study inferred that the sex of DBA/1J mice or Lewis rats did not affect the development of experimental arthritis.
Field-collected samples	The study did not involve samples collected from the field.
Ethics oversight	The animal experiments were performed according to the guidelines for Care and Use of Laboratory Animal Experience of Jilin University and approved by the Institution Animal Ethics Committee of Jilin University (Changchun, China). (SY0208, SY0422, 2021SY0421, 2021SY0422, 2022YNPZSY0617, 2022YNPZSY0618).

Note that full information on the approval of the study protocol must also be provided in the manuscript.

Flow Cytometry

Plots

Confirm that:

- [x] The axis labels state the marker and fluorochrome used (e.g. CD4-FITC).
- [x] The axis scales are clearly visible. Include numbers along axes only for bottom left plot of group (a 'group' is an analysis of identical markers).
- [x] All plots are contour plots with outliers or pseudocolor plots.
- [x] A numerical value for number of cells or percentage (with statistics) is provided.

Methodology

Sample preparation	The RAW264.7 and BMDMs cells were used for the flow cytometry analysis.<br>For apoptosis analysis, the cells were transfected with different nanoparticles for 6 h in serum-free medium and then incubated with fresh 10% FBS containing medium for 48 h. Afterwards, the cells were collected and stained with Annexin V-FITC and PI solutions according to the manufacturer's instruction.<br>For endocytosis mechanism analysis, the cells were pre-treated with different endocytic inhibitors for 30 min, and then transfected with FITC labled-FP/miR-23b nanoparticles for 6 h in serum-free medium. Afterwards, the cells were collected and washed with PBS three times.
Instrument	FACSCalibur flow cytometry system; CytoFLEX flow cytometry system.
Software	CellQuest Pro software v6.0; CytExpert 2.3; Flowjo v10.0.
Cell population abundance	20,000 cells were randomly collected in each experiment.
Gating strategy	For apoptosis analysis, the RAW264.7 cells were firstly gated by FSC/SSC to achieve the intact cells and then gated by Annexin V-FITC/PI channel. Dot plots were separated into four quadrants: live (Annexin V neg, PI neg), early apoptosis (Annexin V pos, PI neg), late apoptosis (Annexin V pos, PI pos), and necrosis (Annexin V neg, PI pos).<br>For endocytosis mechanism analysis, the RAW246.7 cells were firstly gated by FSC/SSC as intact cells and then analyzed by the FITC channel.

- [x] Tick this box to confirm that a figure exemplifying the gating strategy is provided in the Supplementary Information.
